# A Metabolic Widget Adjusts the Phosphoenolpyruvate-Dependent Fructose Influx in *Pseudomonas putida*

**DOI:** 10.1128/mSystems.00154-16

**Published:** 2016-12-06

**Authors:** Max Chavarría, Ángel Goñi-Moreno, Víctor de Lorenzo, Pablo I. Nikel

**Affiliations:** a Systems and Synthetic Biology Program, National Spanish Center for Biotechnology (CNB-CSIC), Madrid, Spain; b Escuela de Química and CIPRONA, Universidad de Costa Rica, San José, Costa Rica; University of California San Diego

**Keywords:** Cra regulator, metabolic memory, PTS sugar transport, *Pseudomonas putida*, glyceraldehyde-3-P dehydrogenase, nutrient shifts, phosphoenolpyruvate

## Abstract

Fructose uptake in the soil bacterium *Pseudomonas putida* occurs through a canonical phosphoenolpyruvate (PEP)-dependent sugar transport system (PTS^Fru^). The logic of the genetic circuit that rules its functioning is puzzling: the transcription of the *fruBKA* operon, encoding all the components of PTS^Fru^, can escape the repression exerted by the catabolite repressor/activator protein Cra solely in the presence of intracellular fructose-1-P, an agonist formed only when fructose has been already transported. To study this apparently incongruous regulatory architecture, the changes in the transcriptome brought about by a seamless Δ*cra* deletion in *P. putida* strain KT2440 were inspected under different culture conditions. The few genes found to be upregulated in the *cra* mutant unexpectedly included *PP_3443*, encoding a bona fide glyceraldehyde-3-P dehydrogenase. An *in silico* model was developed to explore emergent properties that could result from such connections between sugar uptake with Cra and PEP. Simulation of fructose transport revealed that sugar uptake called for an extra supply of PEP (obtained through the activity of PP_3443) that was kept (i.e., memorized) even when the carbohydrate disappeared from the medium. This feature was traced to the action of two sequential inverters that connect the availability of exogenous fructose to intracellular PEP levels via Cra/PP_3443. The loss of such memory caused a much longer lag phase in cells shifted from one growth condition to another. The term “metabolic widget” is proposed to describe a merged biochemical and regulatory patch that tailors a given node of the cell molecular network to suit species-specific physiological needs.

**IMPORTANCE** The regulatory nodes that govern metabolic traffic in bacteria often show connectivities that could be deemed unnecessarily complex at a first glance. Being a soil dweller and plant colonizer, *Pseudomonas putida* frequently encounters fructose in the niches that it inhabits. As is the case with many other sugars, fructose is internalized by a dedicated phosphoenolpyruvate (PEP)-dependent transport system (PTS^Fru^), the expression of which is repressed by the fructose-1-P-responding Cra regulatory protein. However, Cra also controls a glyceraldehyde-3-P dehydrogenase that fosters accumulation of PEP (i.e., the metabolic fuel for PTS^Fru^). A simple model representing this metabolic and regulatory device revealed that such an unexpected connectivity allows cells to shift smoothly between fructose-rich and fructose-poor conditions. Therefore, although the metabolic networks that handle sugar (i.e., fructose) consumption look very similar in most eubacteria, the way in which their components are intertwined endows given microorganisms with emergent properties for meeting species-specific and niche-specific needs.

## Abstract (precis)

The regulatory nodes that govern metabolic traffic in bacteria often show connectivities that could be deemed unnecessarily complex at a first glance. Being a soil dweller and plant colonizer, *Pseudomonas putida* frequently encounters fructose in the niches that it inhabits. As is the case with many other sugars, fructose is internalized by a dedicated phosphoenolpyruvate (PEP)-dependent transport system (PTS^Fru^), the expression of which is repressed by the fructose-1-P-responding Cra regulatory protein. However, Cra also controls a glyceraldehyde-3-P dehydrogenase that fosters accumulation of PEP (i.e., the metabolic fuel for PTS^Fru^). A simple model representing this metabolic and regulatory device revealed that such an unexpected connectivity allows cells to shift smoothly between fructose-rich and fructose-poor conditions. Therefore, although the metabolic networks that handle sugar (i.e., fructose) consumption look very similar in most eubacteria, the way in which their components are intertwined endows given microorganisms with emergent properties for meeting species-specific and niche-specific needs.

## INTRODUCTION

**FIG 1:**
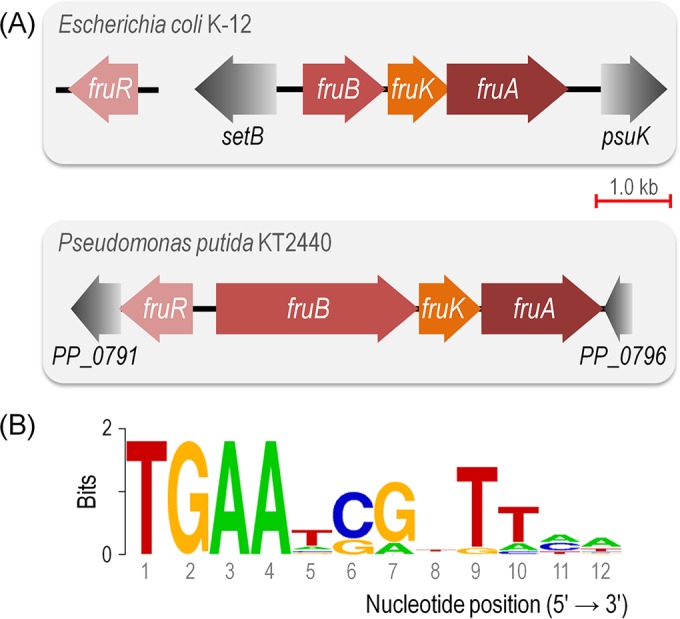
Genomic organization of the *fruBKA* locus in *Escherichia coli* K-12 and *Pseudomonas putida* KT2440 and structure of the Cra binding site. (A) Conserved genomic organization of the *fruBKA* operon in enterobacteria and in *P. putida*. This gene cluster encompasses *fruB* (fructose-specific EI/HPr/EIIA component of the PTS), *fruK* (1-phosphofructokinase), and *fruA* (fructose-specific subunit IIC of the PTS). Note that while the *cra* (i.e., *fruR*) gene lies far away from the *fruBKA* gene cluster in *E. coli*, the corresponding orthologue in strain KT2440 forms part of the same genomic locus but located in a divergent fashion. (B) The canonical *E. coli* consensus sequence for the binding of Cra to DNA was retrieved from the PRODORIC database (53, 54). Note that this sequence forms an incomplete palindrome in which the left half-site is considerably better conserved than the right half-site (the Cra binding sites of *P. putida* examined in this work are closer to a perfect palindrome; see below).

The diversity of nutrient transport mechanisms and the way that their intake is coupled to specific metabolic demands are one of the most puzzling features in environmental bacteria (1–3). While it is tempting to attribute such a diversity and complexity to the mere evolutionary history of the bacteria at stake, some scenarios are conspicuously perplexing and suggest that some adaptive advantage of the corresponding molecular architectures has shaped the cognate uptake processes (4, 5). For instance, glucose (Glu) and fructose (Fru) are both transported in *Escherichia coli* through the general phosphoenolpyruvate (PEP)-dependent sugar transport system (PTS) (6–8). This transport device separately phosphorylates the two sugars as a step prior to their import in a fashion ultimately controlled by the intracellular PEP/pyruvate (Pyr) ratio. In this way, an internal metabolic signal (i.e., the PEP/Pyr ratio) propagates into instructing the PTS to either increase or restrict sugar intake (9). In contrast, the soil bacterium *Pseudomonas putida* KT2440 has a non-PTS transport mechanism for Glu (which can follow either phosphorylation of the hexose or its oxidation to organic acids [10]), while Fru is captured through a dedicated PTS^Fru^ (see Fig. S1 in the supplemental material) (11–13). This Fru-only transport system involves two unique polyproteins: EI/HPr/EIIA^Fru^ (cytoplasmic, encoded by *fruB*) and EIIBC (membrane bound, encoded by *fruA*). Interestingly, in *E. coli* the expression of the *fruBKA* operon is downregulated by FruR (also known as the catabolite repressor/activator [Cra] protein) (14, 15). This repressor protein is considered to be a sensor of glycolytic flux due to its sensitivity to the metabolic intermediate fructose-1,6-P_2_ (FBP) (16) and also because the *cra* gene maps in a chromosomal location far from *fruBKA* (17, 18). In *P. putida*, the same gene complement is arranged as a *fruBKA* operon (19, 20), and its transcription is controlled by the product of the adjacent *fruR*/*cra* gene (Fig. 1A). However, the corresponding Cra^*P. putida*^ protein does not respond to FBP at all but solely to fructose-1-P (F1P) (21, 22), a metabolite that can be formed only upon the action of PTS^Fru^ on Fru. In sum, when the Fru transport systems of *E. coli* and *P. putida* are compared, the same basic constituents are found: *fruB, fruA* (along with a third gene needed for F1P phosphorylation, *fruK*), the Cra repressor, and the phosphorylated forms of Fru (i.e., F1P and FBP) as the effectors. The canonical Cra operator, on the other hand, is well conserved among bacterial species (Fig. 1B) (17, 23). Yet, all these elements appear to be connected differently in terms of functionality, and they seem to respond to different physiological/environmental cues. In fact, the most intriguing aspect of the metabolic architecture in each case is the role of Cra/FruR on the regulation of PTS^Fru^. In enterobacteria, Cra is a transcriptional regulator that controls the expression of a large number of genes in central carbon metabolism (CCM) according to the distribution of glycolytic fluxes (24–26). In contrast, Cra^*P. putida*^ strongly binds an operator at the *P*_*fruB*_ promoter in a fashion exclusively dependent on the intracellular F1P concentration (22). The lack of other Cra^*P. putida*^ targets in the chromosome of strain KT2440 is somewhat intriguing considering the plethora of CCM genes regulated by the orthologue in enterobacteria. Furthermore, the regulatory logic of Fru transport in *P. putida* is a rather perplexing one: F1P is needed to relieve the repression exerted by Cra^*P. putida*^ on the expression of the *fruBKA* operon, and the expression of this operon is obviously needed for the assembly of the PTS^Fru^ complex, without which Fru cannot be transported into the cells. Yet, F1P cannot be generated unless a functional PTS^Fru^ is in place and some Fru has been transported to the cytoplasm.

10.1128/mSystems.00154-16.1 Figure S1  Central carbon metabolism in *Pseudomonas putida* KT2440. Representation of glucose, fructose, and succinate metabolism in *P. putida* KT2440. Individual components of different metabolic blocks within central carbon metabolism are highlighted with different colors as indicated in the lower corner to the left. The abbreviations for the metabolites and enzymes in this metabolic map are as follows: Glk, glucokinase; G6P, glucose-6-P; 6PG, 6-phosphogluconate; KDPG, 2-keto-3-deoxy-6-phosphogluconate; GnuK, gluconokinase; Gcd, glucose dehydrogenase; FBP, fructose-1,6-P_2_; Fbp, fructose-1,6-bisphosphatase; Pgi, G6P isomerase; Zwf, G6P dehydrogenase; Pgl, 6-phosphogluconolactonase; KguK, 2-ketogluconate kinase; KguD, 2-ketogluconate-6-P reductase; Edd, 6PG dehydratase; EDa, KDPG aldolase; Fda, FBP aldolase; TpiA, triose phosphate isomerase; Gnd, 6PG dehydrogenase; PPs, pool of pentose phosphate; Rpe, ribulose-5-P 3-epimerase; TktA, transketolase; Tal, transaldolase; RpiA, ribose-5-P isomerase; GA3P, glyceraldehyde-3-P; BPG, 1,3-P_2_-glycerate; 3PG, glycerate-3-P; 2PG, glycerate-2-P; Pgk, phosphoglycerate kinase; Pgm, phosphoglycerate mutase; Eno, phosphopyruvate hydratase; PEP, phosphoenolpyruvate; AceEF, LpdGV, Lpd3, and AcoA, pyruvate dehydrogenase; MaeB, malic enzyme; Pyk, pyruvate kinase; Mdh, malate dehydrogenase; Mqo, malate:quinone oxidoreductase; AccC-2, pyruvate carboxylase subunit A; OadA, pyruvate carboxylase subunit B; Ppc, PEP carboxylase; FumC, fumarate hydratase; SdhABCD, succinate dehydrogenase; SucACD and KgdB, succinyl-coenzyme A synthetase and 2-ketoglutarate dehydrogenase; AcnAB, aconitate hydratase; Icd, isocitrate dehydrogenase; GltA, citrate synthase; FruB/FruA, PTS^Fru^; and FruK, 1-phosphofructokinase. Note that the metabolic node connecting GA3P with downward metabolism is indicated by highlighting the four GA3P dehydrogenase enzymes of strain KT2440. The two key metabolites involved in the metabolic widget described in this work (i.e., GA3P and PEP) are indicated in blue. Download Figure S1, PDF file, 0.3 MB. Copyright © 2016 Chavarría et al. 2016 Chavarría et al. This content is distributed under the terms of the Creative Commons Attribution 4.0 International license.

The work described in this contribution was set to clarify such a metabolic conundrum, i.e., why PTS^Fru^ of *P. putida* follows such an apparently incongruous regulatory layout. To this end, the consequences of seamlessly deleting *cra* on the cognate whole-genome transcriptome were examined in *P. putida* KT2440 growing under different metabolic regimens. As shown below, a key gene (*PP_3443*) encoding a glyceraldehyde-3-P (GA3P) dehydrogenase (GAPDH) activity was found to be regulated by Cra^*P. putida*^. This new regulatory connection grants Cra^*P. putida*^ the control of the overall GAPDH activity through PP_3443, thereby regulating the levels of PEP depending on metabolic requirements. Yet, what is the impact of this regulatory pattern on the cell physiology? By modeling a combined metabolic and regulatory circuit *in silico* that emerges from such a new connection, it was discovered that Cra^*P. putida*^ regulates the availability of PEP (and therefore the uptake of Fru) through PP_3443. Also, the resulting device (which we have called a “metabolic widget”) acts as a memory patch that eases the shift of cells between conditions with different levels of Fru availability. Thus, new and often subtle regulatory connections adjust default and widely shared metabolic networks to specific needs of given bacteria in particular environmental niches. New functionalities in biochemical setups thereby emerge not because of the mere enumeration of the components at stake but owing to novel functional relationships between these elements.

## RESULTS AND DISCUSSION

### Transcriptional analysis of wild-type *P. putida* KT2440 and its Δ*cra* derivative reveals unexpected regulatory targets.

Previous studies have indicated that the Cra protein of *E. coli* and *Salmonella enterica* serovar Typhimurium acts as a dual regulator, as it represses the expression of some CCM genes (e.g., *pfkA*, *pykA*, *pykF*, *acnB*, *edd*, *eda*, *mtlADR*, and *gapB*) and activates the expression of others (e.g., *ppsA*, *fbp*, *pckA*, *acnA*, *icd*, *aceA*, and *aceB*) (27–29). As a physiological consequence of the regulation exerted by Cra on multiple genes of CCM in *E. coli*, this regulator controls the carbon flow and ultimately influences the utilization of several substrates (30–32).

The genome of the soil bacterium *P. putida* KT2440 encodes a Cra orthologue whose gene (*cra*, also called *fruR* [Fig. 1A]) is divergently oriented in respect to the *fruBKA* operon. Cra^*P. putida*^ binds one quasipalindromic operator (5′-TTA AAC ⋅ GTT TCA-3′, Fig. 1B) located between *P*_*fruB*_ and *fruB,* thereby repressing the expression of the entire *fruBKA* operon in an F1P-dependent fashion. Because other possible regulatory functions for Cra^*P. putida*^ remain unknown, the genome-wide transcription landscapes of wild-type and Δ*cra* cells grown on different carbon sources (Glu, succinate [Suc], and Fru) were compared by means of deep RNA sequencing (33, 34). We first focused on the transcription of genes encoding components of CCM at the single-nucleotide resolution. In agreement with the results of previous studies, differences were observed in the transcription of the *fruBKA* operon between wild-type KT2440 and the Δ*cra* mutant (see Fig. S2 in the supplemental material). No transcription was observed in Glu- or Suc-grown wild-type cells, while *fruBKA* was highly transcribed in the Δ*cra* background (i.e., Cra^*P. putida*^ represses the expression of components of PTS^Fru^). Wild-type cells grown on Fru represent a control for the results obtained in Glu and Suc as F1P is freely produced under this condition, it binds to Cra^*P. putida*^, and the transcription of PTS^Fru^ is derepressed (see Fig. S2 in the supplemental material). Yet, what is the situation for other genes of CCM?

10.1128/mSystems.00154-16.2 Figure S2  Sequence coverage plots for the *fruBKA* gene cluster of *Pseudomonas putida* KT2440 and its Δ*cra* derivative analyzed at the single-nucleotide level. Data correspond to the expression pattern for samples taken during the mid-logarithmic phase of growth in cultures developed on glucose (Glu), fructose (Fru), or succinate (Suc) as the sole carbon source. WT, wild-type strain. Download Figure S2, PDF file, 0.1 MB. Copyright © 2016 Chavarría et al. 2016 Chavarría et al. This content is distributed under the terms of the Creative Commons Attribution 4.0 International license.

**FIG 2:**
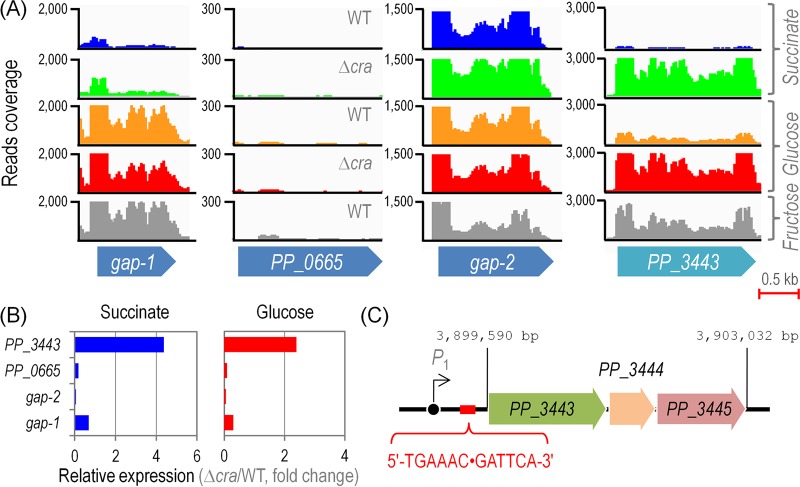
Sequence coverage plots for genes encoding glyceraldehyde-3-P dehydrogenase activities in *Pseudomonas putida* KT2440 and its Δ*cra* mutant analyzed at the single-nucleotide level. (A) Coverage plots for the raw deep RNA sequencing data. The plots shown in the diagram correspond to the expression pattern of the selected genes in samples taken during mid-log phase from cultures developed on glucose, fructose, or succinate as the sole carbon source as indicated at the far right. (B) Quantitative analysis of gene expression for the four genes encoding glyceraldehyde-3-P dehydrogenase activities. The bars represent the mean value of the expression level for each gene observed in the Δ*cra* mutant normalized to the expression level detected in *P. putida* KT2440, expressed as −log_2_(Δ*cra*/wild type). Differences in the pairwise comparison of the expression of *PP_3443* against that of any other gene in the diagram (i.e., *gap-1*, *gap-2*, or *PP_0665*) were significant as judged by the corresponding false discovery rate values (51). (C) The regulatory region of the *PP_3443* gene in *P. putida*, containing a single operator identified as indicated in the outline. The sequence 5′-TGA AAC GAT TCA-3′ (highlighted in red) corresponds to the quasipalindromic Cra binding motif upstream of the *PP_3443* gene. WT, wild-type strain.

No gene related to CCM in *P. putida* KT2440, belonging to the pathways depicted in Fig. S1 in the supplemental material, seemed to be transcriptionally regulated by Cra^*P. putida*^ across the culture conditions tested in this study (data not shown), with the intriguing exception of *PP_3443*. The expression of *PP_3443* was repressed in Suc and Glu and transcribed in strain KT2440, but this pattern changed in the Δ*cra* mutant, in which the gene was highly transcribed under either gluconeogenic or glycolytic growth conditions (Fig. 2A). Since PP_3443 has been recently shown to represent a GAPDH in *P. putida* growing on different substrates (35), we asked the question whether the transcription of other genes known to encode GAPDH activities could be also under the control of Cra^*P. putida*^ (Fig. 2A). *PP_0665* was the only GAPDH-encoding gene that did not show any significant level of transcription under all the conditions tested. The gene encoding Gap-1, the main glycolytic GAPDH activity, was expectedly transcribed in Glu- and Fru-grown cells (but not on Suc) irrespective of the presence of Cra^*P. putida*^. In contrast, *gap-2* was highly transcribed across the culture conditions and strains tested, and therefore, its expression seems to be constitutive. When these transcription data were individually analyzed and converted into the corresponding relative expression levels, the effect of Cra^*P. putida*^ on the transcription of *PP_3443* became even more evident (Fig. 2B). In particular, the level of expression of this gene increased by 4.4- and 2.4-fold in the Δ*cra* mutant compared to the wild-type strain in Suc and Glu cultures, respectively.

This regulatory pattern on the *PP_3443* gene was further checked *in silico* by applying the Cra binding *E. coli* sequence consensus (Fig. 1B) to the 5′ untranslated region of *PP_3443*. Further, the entire genome of strain KT2440 was scanned to check for potential regulatory targets. Possible Cra^*P. putida*^ binding sites were identified in the intergenic region of three CCM genes (i.e., *gap-1*, 5′-TGA AAC ⋅ GGT TTT-3′; *edd*, 5′-TGA AAC ⋅ GGT TTT-3′; and *PP_3443*, 5′-TGA ATC ⋅ GTT TCA-3′) (Fig. 2C; see also Table S1 in the supplemental material). Therefore, deep sequencing of RNA transcripts and the analysis of possible Cra^*P. putida*^ operators in the genome of *P. putida* KT2440 indicated that the only bona fide CCM gene subjected to transcriptional regulation by Cra^*P. putida*^ is *PP_3443*, in addition to the known regulation on the Fru utilization operon. Again, this information reveals that the regulatory tasks of Cra^*P. putida*^ are entirely different with respect to enterobacteria.

10.1128/mSystems.00154-16.6 Table S1  Hypothetical DNA binding sites of the Cra protein in the chromosome of *Pseudomonas putida* KT2440 obtained with the PRODORIC database. Download Table S1, PDF file, 0.05 MB. Copyright © 2016 Chavarría et al. 2016 Chavarría et al. This content is distributed under the terms of the Creative Commons Attribution 4.0 International license.

### Cra regulates the GAPDH activity of *P. putida* KT2440.

**FIG 3:**
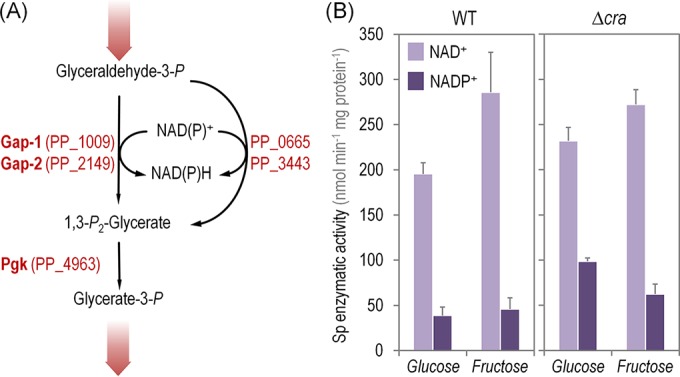
*In vitro* determination of the glyceraldehyde-3-P dehydrogenase activity in *Pseudomonas putida* KT2440 and its Δ*cra* mutant. (A) Biochemical sequence proposed for the processing of glyceraldehyde-3-P. Enzymes involved in these conversions are shown beside the reaction that they catalyze. Note that the first biochemical step is catalyzed by the glyceraldehyde-3-P dehydrogenases (Gap-1 and Gap-2) and the two other isoforms of these enzymes, encoded by *PP_0665* and *PP_3443*. All the reactions are conventionally written in the catabolic direction, and the wide shaded arrows indicate the connection of this series of biochemical reactions with the rest of the central carbohydrate metabolic pathways. (B) *In vitro* quantitation of the total specific (Sp) activity of glyceraldehyde-3-P dehydrogenase in *P. putida* KT2440 and its isogenic Δ*cra* derivative determined in cells grown on glucose or fructose and using different redox cofactors as indicated. Each bar represents the mean value of the corresponding enzymatic activity ± standard deviation of duplicate measurements from at least three independent experiments. WT, wild-type strain.

Since the only gene in central CCM over which we observed transcriptional differences between wild-type *P. putida* and the Δ*cra* mutant was *PP_3443*, the next relevant question was whether this transcriptional pattern correlates with an actual metabolic regulation. As indicated above, the GA3P metabolic node in *P. putida* KT2440 comprises four potential enzymes (Fig. 3A). The *in vitro* evaluation of the total GAPDH activity in a *P. putida* Δ*PP_3443* strain grown on glycerol indicated that this enzyme seems to be NADP^+^ dependent (35). Based on these previous studies and the RNA sequencing results discussed above, we measured the total GAPDH activity in wild-type cells and in the Δ*cra* mutant. The *in vitro* enzymatic assays were performed using either NAD^+^ or NADP^+^ as the cofactor in cell extracts obtained from cells grown on Glu or Fru.

The GAPDH activities with either redox cofactor were similar in the wild-type strain and its Δ*cra* derivative when Fru was used as the carbon source (Fig. 3B). As previously explained for the expression of components of PTS^Fru^, the regulatory functions of Cra^*P. putida*^ are deactivated in Fru, as the effector F1P is continuously produced and derepression occurs constitutively. Although in a Δ*cra* mutant the expression of *PP_3443* is fully derepressed, no significant differences were observed in enzymatic assays between the two strains. Results from cells grown on Glu as the sole carbon source, however, clearly evidenced the regulation exerted by Cra on *PP_3443*. The *in vitro* assays revealed a 1.22-fold increase in the NAD^+^-dependent GAPDH activity between the wild type and the Δ*cra* mutant, and far more clear differences were observed in the GAPDH activity evaluated in the presence of NADP^+^ as the cofactor (i.e., a 1.54-fold increase in the specific GAPDH activity in the Δ*cra* mutant with respect to *P. putida* KT2440). In a similar fashion as the results of transcriptomic analysis previously discussed, the direct comparison of the specific enzymatic activities of the wild-type strain grown on Glu and Fru represents a useful control for the observations obtained in the Δ*cra* mutant cells. This regulation was disclosed by the 1.46-fold-higher specific GAPDH activity observed in *P. putida* KT2440 grown in Fru than in the same cells grown on Glu (Fig. 3B). Thus, the results of *in vitro* enzymatic determinations certify the regulation of Cra^*P. putida*^ on the overall GAPDH activity that the transcriptional evidence had suggested. Yet, what is the physiological relevance of this delicate control in the GA3P node mediated by Cra^*P. putida*^ in strain KT2440?

### The regulation exerted by Cra^*P. putida*^ on *PP_3443* is related to phosphate availability for the operation of PTS^Fru^.

The transcriptomic and enzymatic data established a connection between Cra^*P. putida*^ and the overall GAPDH activity through the product encoded by *PP_3443*. This observation raises new questions: why would *P. putida* require a higher GAPDH activity when growing on Fru than when growing on Glu or Suc? And, more importantly, what are the physiological consequences of this catabolic regulation? The close examination of the pathways involved in CCM in *P. putida* KT2440 (see Fig. S1 in the supplemental material) provides a hint to answer these questions. GAPDH enzymes are located in a key regulatory point of CCM: the GA3P node. This key metabolite can follow several central routes, i.e., (i) GA3P can be directed toward the formation of PEP and Pyr to subsequently enter into the tricarboxylic acid (TCA) cycle as acetyl coenzyme A; (ii) it can be recycled upward for the production of FBP, fructose-6-P, and glucose-6-P to generate reducing power (namely, NADPH + H^+^ [36]) through the so-called EDEMP cycle (11); and (iii) it may be also used in reversible reactions within the pentose phosphate pathway. GA3P is therefore a very important metabolic hub from where carbon fluxes can be accommodated through several branching pathways according to biochemical and environmental requirements. However, there is another factor that needs to be considered in this picture: the phosphate group borne by GA3P.

As mentioned in the introduction, PTS^Fru^ of *P. putida* (composed of FruA/EIIBC and FruB/EI-HPr-EIIA) coexists with PTS^Ntr^ (composed of PtsP/EI^Ntr^, PtsO/NPr, and PtsN/EIIA^Ntr^). The source of the high-energy phosphate residue that fuels the activity of both PTS systems is PEP (37). Since the source of such a phosphate residue needed for the conversion of Fru into F1P is PEP (12), and considering that this metabolite can be generated from GA3P only under glycolytic conditions (i.e., using either Glu or Fru as the carbon source) (38), the interplay between carbon fluxes and the availability of high-energy phosphate residues was also considered in our interpretation. In particular, we hypothesized that the overall GAPDH activity, from which PEP is ultimately obtained, rules the availability of high-energy phosphate needed for Fru phosphorylation through PTS^Fru^. Since Cra^*P. putida*^ controls the expression of *PP_3443* in strain KT2440 and therefore the total GAPDH activity, it seems plausible that this regulation results in differences in the operativity of the whole PTS^Fru^. This hypothesis fits with the distribution of metabolic fluxes, based on ^13^C tracers, in wild-type *P. putida* KT2440 when cells are grown on Glu and Fru (see Fig. S3 in the supplemental material). When the normalized metabolic fluxes from GA3P to PEP were evaluated, a 25% increase was observed in the GAPDH flux in Fru-grown cells over the flux in those grown in Glu. From a more general perspective, the normalized fluxomic data indicated that the net fluxes connecting GA3P → glycerate-3-P → PEP → Pyr are higher in Fru- than in Glu-grown *P. putida* cells.

10.1128/mSystems.00154-16.3 Figure S3  Net metabolic fluxes around the glyceraldehyde-3-P node of wild-type *Pseudomonas putida* grown on glucose and fructose. The value of each metabolic flux in this subnetwork was normalized to the specific carbon source uptake rate (arbitrarily set to 100; actual *q*_S_ values are also given in the sketch) for wild-type *P. putida* growing on either glucose (A) or fructose (B), and only the fluxes connecting glyceraldehyde-3-P to pyruvate are shown in this sketch. Note that the reactions connecting these metabolites with the rest of the metabolic network are indicated by means of wide shaded arrows. The reaction catalyzed by glyceraldehyde-3-P dehydrogenase is shown in red along the four isozymes responsible for this transformation in strain KT2440. CDW, cell dry weight. Download Figure S3, PDF file, 0.1 MB. Copyright © 2016 Chavarría et al. 2016 Chavarría et al. This content is distributed under the terms of the Creative Commons Attribution 4.0 International license.

### Kinetic modeling of the fructose intake in *P. putida* KT2440 reveals a dynamic behavior in central carbon metabolism ruled by Cra^*P. putida*^.

**FIG 4:**
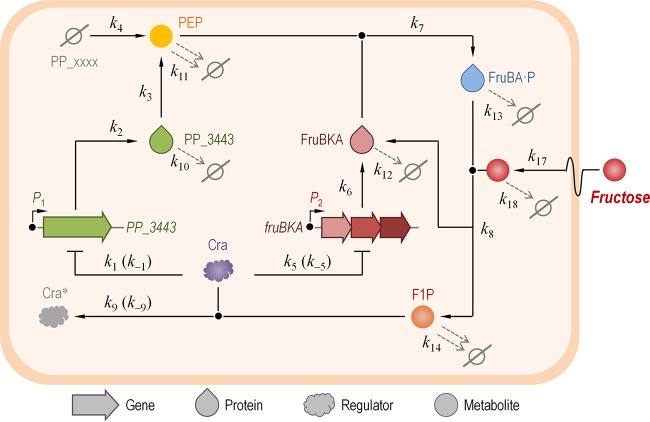
Network used for modeling the dynamics of fructose uptake and the regulation exerted by Cra in *Pseudomonas putida* KT2440. The model merges the dynamics of gene expression, protein stability, enzymatic activity, and the concentration of intracellular and extracellular metabolites. Note that this sketch does not incorporate all the possible connections existing in the system; rather, it depicts only those reactions deemed responsible for the dynamics of the network considered and thus targeted for the simulation studies described here. The values of all the *k* constants (representing individual rates in the model) are indicated in Table 1. F1P, fructose-1-P; PEP, phosphoenolpyruvate; Cra*, inactive form of the Cra regulator; PP_xxxx, alternative glyceraldehyde-3-P dehydrogenases (i.e., other than PP_3443). The Ø symbol represents degradation of the corresponding species.

The transcriptomic, fluxomic, and quantitative physiology data above point toward a connection between the Cra^*P. putida*^ regulator, the activity of GAPDH (and hence the intracellular availability of PEP), and the process of Fru uptake in *P. putida* KT2440. Yet, these functional relationships are of a purely qualitative nature, and therefore, the next objective was to analyze these connections with methods that go beyond experimental techniques to describe the process of Fru uptake mediated by PTS^Fru^ in this bacterium. The functional connections between the elements at stake are so specific and ephemeral that is extremely difficult to evaluate them *in vivo*. In order to fulfill this objective, we built a mathematical model of these cell components and kinetic parameters that reveals the details of short-lived dynamics (Fig. 4). The resulting dynamic model takes into consideration (and merges) (i) the transcriptional regulation exerted by Cra^*P. putida*^ on *PP_3443* and *fruBKA*, (ii) the actual GAPDH activity brought about by PP_3443 and the transporter activity of FruAB/FruK (i.e., PTS^Fru^), (iii) the intracellular concentrations of PEP and F1P, and (iv) the extracellular levels of Fru as a substrate. Furthermore, the regulatory effect of F1P on Cra^*P. putida*^ was also taken into account as a variable in the model. Note, however, that the sketch shown in Fig. 4 does not incorporate all the potential connections that exist in the system; instead, it describes those reactions considered responsible for the dynamics of the entire network under study. These were the relevant parameters targeted for simulation studies. In particular, the intracellular concentration of PEP was used as a measuring reference (i.e., a proxy) in both wet experiments and *in silico* analysis. The reactions corresponding to the model circuit are indicated in Fig. S4A in the supplemental material, and the rate values along with initial species concentrations are indicated in Table 1. Species dynamics over time were simulated deterministically with a set of coupled ordinary differential equations (ODEs) (see Fig. S4B in the supplemental material). The selection of rates and molecular species intervening in the model was done on the basis of (i) direct measurements in wet-lab experiments whenever possible (e.g., *k*_17_, which represents the specific rate of Fru uptake, *q*_S_) and (ii) estimation of some parameters taken from the literature when direct experimental values were not available (e.g., *k*_1_, which represents the affinity of Cra^*P. putida*^ for the *P*_1_ promoter that drives the expression of *PP_3443*). In any case, the reconstructed network and the corresponding model suggest that the alteration of the set of estimated values would lead to relatively small quantitative changes in the overall output (as opposed to qualitative changes). The ODEs were coded using Python as the programming language.

**FIG 5:**
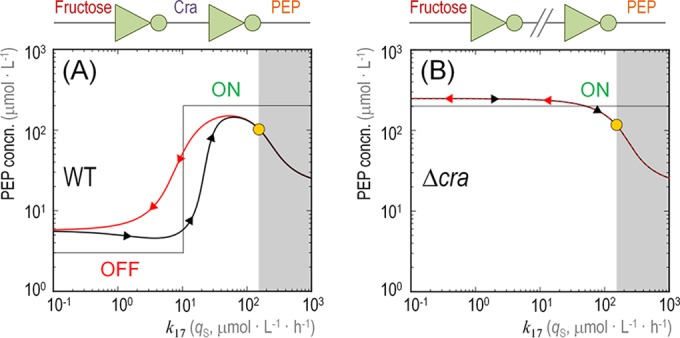
Dynamic modeling of the intracellular availability of phosphoenolpyruvate with respect to the specific rate of fructose uptake. The plots indicate the level of phosphoenolpyruvate (PEP, *y* axis) while varying the specific rate of fructose uptake (*q*_S_, *k*_17_ in the network shown in Fig. 4, *x* axis) for the wild-type (WT) *Pseudomonas putida* KT2440 strain (A) and its Δ*cra* derivative (B). Black lines (arrows pointing to the right) show the state of PEP in the system while *k*_17_ increases from an initial state without fructose in the medium (far left). Red lines (arrows pointing to the left) show the state of PEP in the system while *k*_17_ decreases from the final state (far right). The OFF and ON states of the system are indicated in the graphics, and the architecture of the network is shown on top of the plots in terms of inverters (connecting PEP and fructose through the regulatory action exerted by Cra). The yellow dot indicates the experimentally measured values for the intracellular PEP level and *k*_17_. The shadowed areas in the plots indicate what would happen if the cells were hypothetically overloaded with fructose (i.e., beyond the physically possible limits determined experimentally). As shown in the diagrams, the level of available PEP would decrease, as it would be consumed right away to mediate fructose transport.

10.1128/mSystems.00154-16.4 Figure S4  Reactions used in the kinetic model and ordinary differential equations utilized for the calculations. The kinetic model proposed in Fig. 5 in the main text is composed of a series of reactions (A) that were interpreted by means of a set of ordinary differential equations (B). In the model, *k*_1_ and *k*_−1_ represent the binding and unbinding rates, respectively, of the Cra regulator to promoter *P*_1_ (controlling the expression of *PP_3443*). They were estimated to have the same value as *k*_5_ and *k*_−5_, experimentally measured in a previous work, and they represent the binding and unbinding rates, respectively, of the Cra regulator to promoter *P*_2_ (controlling the expression of *fruBKA*). The *k*_2_ parameter merges transcription and translation into a single event (represented as such for the sake of simplicity). Rates *k*_3_ and *k*_4_ describe the production of phosphoenolpyruvate (PEP) from either PP_3443 or other sources, respectively. These values were adjusted to experimental observations. The *k*_6_ parameter represents the transcription and translation of *fruBKA*. Rates *k*_7_ and *k*_8_ represent 2:1 reactions; the former indicates the formation of FruBKA-P, and the latter represents the generation of fructose-1-P (F1P). Finally, *k*_9_ and *k*_−9_ are the constants for binding/unbinding of F1P and the Cra regulator. Besides these parameters, two different sets of degradation constants were defined, the first set for proteins and the second one for metabolites. The rates for the first group (*k*_10_, *k*_12_, and *k*_13_ in the sketch) are lower than the ones for the second (*k*_11_, *k*_14_, and *k*_18_ in the sketch), as metabolites are degraded much faster than proteins. Finally, rate *k*_17_ represents fructose uptake and was measured experimentally (i.e., *q*_S_). As for the initial values for the state of the promoters in the model (*P*_1_ and *P*_2_), they were estimated from this work; their state with the regulator bound [*P*_(r)1_ and *P*_(r)2_] was set to be 0 at the beginning of the simulation. Note that neither the free Cra protein nor its repressed complex (Cra^r^, bound to F1P) is subjected to degradation rates. This is because they are not subjected to production either; these reactions were deleted for the sake of simplicity (it is only the presence or absence of Cra that matters in the model and not its production dynamics). The state of all the rest of the individual species was initially set to 0, representing an initial pristine state (Table 1 contains further details). Download Figure S4, PDF file, 0.1 MB. Copyright © 2016 Chavarría et al. 2016 Chavarría et al. This content is distributed under the terms of the Creative Commons Attribution 4.0 International license.

**TABLE 1:** Rates, values, and species used for simulation

Rate^b^	Value and unit	Source^a^
*k*_1_	1 μmol^−1^ ⋅ liter ⋅ h^−1^	Es; this work
*k*_−1_	50 × 10^−3^ h^−1^	Es; this work
*k*_2_	60 h^−1^	Es; 59, 60
*k*_3_	8 × 10^4^ h^−1^	Ft; this work
*k*_4_	10 μmol^−1^ ⋅ liter^−1^ ⋅ h^−1^	Ft; this work
*k*_5_	1 μmol^−1^ ⋅ liter ⋅ h^−1^	Ob; this work
*k*_−5_	50 × 10^−3^ h^−1^	Ob; this work
*k*_6_	90 h^−1^	Es; 59, 60
*k*_7_	208 μmol^−1^ ⋅ liter ⋅ h^−1^	Es; 59, 60
*k*_8_	30 μmol^−1^ ⋅ liter ⋅ h^−1^	Es; 59, 60
*k*_9_	0.35 μmol^−1^ ⋅ liter ⋅ h^−1^	Ob; this work
*k*_−9_	50 × 10^−3^ h^−1^	Ob; this work
*k*_10_	2 h^−1^	Es; 59, 60
*k*_11_	17 h^−1^	Es; this work
*k*_12_	0.17 h^−1^	Es; 59, 60
*k*_13_	0.17 h^−1^	Es; 59, 60
*k*_14_	17 h^−1^	Es; this work
*k*_17_	168 μmol ⋅ liter^−1^ ⋅ h^−1^	Ob; this work
*k*_18_	1.7 h^−1^	Es; this work
*P*_1_	1.7 × 10^−3^ μmol ⋅ liter^−1^	Ob; this work
*P*_2_	1.7 × 10^−3^ μmol ⋅ liter^−1^	Ob; this work
Cra	4 μmol ⋅ liter^−1^	Es; this work

a All the parameters are codified as Es (estimated), Ob (experimentally obtained), or Ft (fitted).

b See the model in Fig. 4 and the legend to Fig. S4 in the supplemental material for information on the rates.

Figure 5 shows the results of a simulation in which the PEP concentration was plotted as a function of the specific rate of Fru uptake (i.e., *q*_S_, represented in the model by *k*_17_). The first indication of the dynamics of the system came from the shape of the trajectories representing PEP concentration, which differed depending on whether the *q*_S_ values were increasing or decreasing. In other words, there was a hysteresis process taking place in wild-type *P. putida* KT2440 cells at low-to-medium *q*_S_ values whereby, for a given *x* axis point, there were two very different values of the *y* axis depending on whether the system was transiting from a high *q*_S_ value (going from right to left in the *x* axis) or from a low *q*_S_ value (going from left to right in the *x* axis) (Fig. 5A). Such a hysteresis process disappeared altogether in the Δ*cra* mutant (Fig. 5B). Therefore, this behavior, affecting the internal state of the cells in terms of PEP availability, stems from the history of the system. The entire network in the wild-type cell acts as a circuit with two inverters in a row, what we purposely call a “metabolic widget”. If the Fru level in the external medium is negligible (and thus *q*_S_ is very low), the output (the actual intracellular PEP concentration) is OFF; if, on the contrary, Fru is present in the culture medium, the output is ON. The circuit is cut off in the Δ*cra* mutant, yielding an always-ON behavior with respect to PEP formation. In both cases, the PEP concentration decreases on the far right because all the components of the system are ON and therefore PEP gets consumed right away to mediate Fru transport, without even transiting through an available state. The hysteresis process is observed in wild-type cells and not in the Δ*cra* mutant derivative because the forces at play in the whole network are rooted in slow dynamics (please refer to the differences highlighted in Fig. S5 in the supplemental material), caused by gene expression/repression events (i.e., as opposed to fast changes in the intracellular metabolite concentration). Since these changes are brought about by gene expression patterns, the behavior of the entire system can be traced to the regulatory action of the Cra^*P. putida*^ protein. The hysteresis phenomenon represents a temporal memory (i.e., the system tends to stay in its previous state). The next standing question was to analyze if PP_3443 participates in such a regulatory circuit.

10.1128/mSystems.00154-16.5 Figure S5  Dynamic modeling of the intracellular availability of some molecular species in the metabolic widget. (Top row) Phosphoenolpyruvate (PEP) and fructose-1-P (F1P) levels over time in the wild-type (WT) cell (top left) and in the Δ*cra* mutant (top right). The levels at the start and end of the graphs are similar, as observed *in vivo*, but the transitions between different states fluctuate substantially. The instability pointed out by the shaded region (top left) is due to the positive feedback of F1P over PEP via the inhibition exerted by Cra^*P. putida*^. Therefore, in the Δ*cra* mutant (top right) such an event is not observed. (Bottom left) The same time course simulation was repeated, but in this case, the promoter upstream of *cra* is monitored in its two possible states: *P*_1_, promoter without Cra^*P. putida*^ bound to the corresponding operator, and *P*_1_(r), promoter with Cra^*P. putida*^ bound in the corresponding operator (note that the sum of the two states is always constant). (Bottom right) Interaction loop. Relationship between promoter *P*_1_ (when Cra^*P. putida*^ is not bound to the operator sequence, *x* axis) and the concentration of PEP (*y* axis). Initially, *P*_1_ is free of Cra^*P. putida*^ (far right in the *x* axis), which is why the PEP concentration is rapidly increasing (going up in the *y* axis). Cra^*P. putida*^ starts repressing *P*_1_ (thus going left on *x* axis), and as a consequence, the level of PEP decreases (going down in the *y* axis). Finally, F1P represses Cra^*P. putida*^; therefore, *P*_1_ is free again and the concentration of PEP increases up to the experimentally measured value (yellow dot). Download Figure S5, PDF file, 0.1 MB. Copyright © 2016 Chavarría et al. 2016 Chavarría et al. This content is distributed under the terms of the Creative Commons Attribution 4.0 International license.

### Hysteresis in the system depends on the activity borne by the Cra^*P. putida*^-regulated *PP_3443* gene.

**FIG 6:**
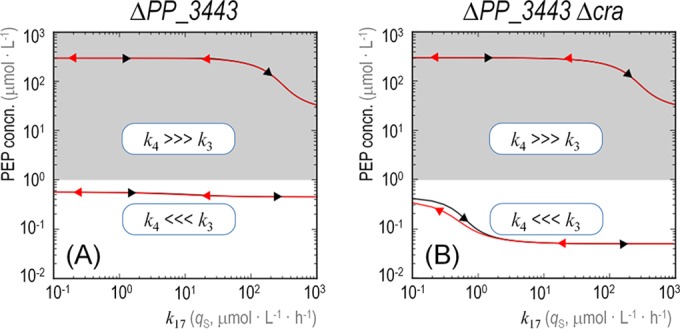
Role of PP_3443 in the dynamic behavior of the metabolic widget and the availability of phosphoenolpyruvate. The plots indicate the level of phosphoenolpyruvate (PEP, *y* axis) while varying the specific rate of fructose uptake (*q*_S_, *k*_17_ in the network shown in Fig. 4, *x* axis) for a Δ*PP_3443* mutant of *Pseudomonas putida* KT2440 (A) and a Δ*PP_3443* Δ*cra* double mutant (B). The lines within the shaded regions on top of each diagram (i.e., when the PEP concentration is >1 μmol ⋅ liter^−1^) correspond to separate simulations where *k*_4_ is much higher than *k*_3_, which represents a higher flow to PEP formation catalyzed by the three remaining glyceraldehyde-3-P enzymes (i.e., other than PP_3443). The bottom lines, in contrast, follow the initial conditions (where *k*_3_ is much higher).

In order to evaluate the role of PP_3443, the GAPDH activity under transcriptional control by the Cra^*P. putida*^ protein, we repeated the simulations described above for a *P. putida* Δ*PP_3443* mutant and a double Δ*PP_3443* Δ*cra* mutant (Fig. 6). In either case, the absence of *PP_3443* means that PEP should be synthesized via the intermediates generated by the three other GAPDH activities of strain KT2440 (or at least Gap-1 and Gap-2, since these are the two variants apparently active as indicated by the transcriptomic data presented above). In terms of the model evaluated here, this translates into *k*_4_ >>> *k*_3_, *k*_4_ and *k*_3_ being the rate of PEP generation through the GAPDH activity of Gap-1 and Gap-2 and that of PP_3443, respectively. When PP_3443 is eliminated from the system (in both Fig. 6A and B), PEP formation expectedly drops drastically at the initial values of *q*_S_ (i.e., the intracellular PEP concentration remains at <1 μmol ⋅ liter^−1^; see white bottom regions in the plots). The lines representing PEP concentration in the shaded regions of the plots look alike, irrespective of the presence of Cra^*P. putida*^, under the assumption that the overall GAPDH activity of the Δ*PP_3443* mutant is enough to provide the PEP needed for Fru uptake and proper cell functioning. In other words, the dynamics of the system described above are completely lost if PP_3443 is absent. In the case of the Δ*cra* mutant, the intracellular levels of PEP decrease further because this metabolite is being used (and consumed) by FruBA. In such a hypothetical scenario, an increase in *k*_4_ (shaded top regions of plots) restores the levels of PEP, which indicates that the routes responsible for PEP production are dynamically adjusted in the cells. No hysteresis happens in the system under these conditions, as no expression/repression events take place and slow reaction dynamics are altogether eliminated.

### Functional validation of the metabolic widget for fructose utilization in *P. putida* KT2440.

**FIG 7:**
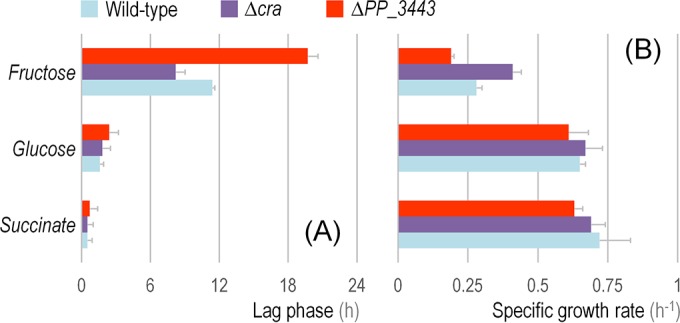
Kinetic and growth parameters of *Pseudomonas putida* KT2440 and its Δ*cra* and Δ*PP_3443* derivatives. Batch cultures of the strains under study, using different gluconeogenic and glycolytic carbon sources as indicated in the plots, were analyzed by the duration of the characteristic lag phase (A) and the specific growth rate of the cells (B). The extension of the lag phase was analytically obtained from growth parameters as detailed by Dalgaard and Koutsoumanis (43), and the specific growth rate was determined during exponential growth. Each bar represents the mean value of the corresponding parameter ± standard deviation of triplicate measurements from at least three independent experiments.

The importance of the regulatory control displayed by the Cra^*P. putida*^/PP_3443/PEP widget described here was made evident when growth parameters of *P. putida* KT2440 and the Δ*cra* and ΔPP_3443 mutant derivatives were analyzed in quantitative physiology experiments (Fig. 7). When using Glu or Suc as the carbon source, the three strains followed rather similar growth patterns, and the differences in the kinetic parameters analyzed were not significant. In fact, the specific growth rates/characteristic doubling times and the duration of the lag phase in these cultures were consistent with previous findings (11, 35, 39), Suc promoting the fastest growth among the conditions tested. A different situation, however, was noted when Fru was used as the substrate. The *P. putida* Δ*cra* mutant, for instance, displayed a remarkable behavior in these Fru-dependent cultures, characterized by (i) a 30% shorter lag phase (Fig. 7A) and (ii) a 46% higher specific growth rate (Fig. 7B) with respect to the wild-type strain. In the wild-type strain, the derepression of the genes encoding the PTS^Fru^ involves the buildup of enough F1P and the binding of this metabolite to Cra^*P. putida*^, thereby bringing about a conformational change in the protein to break free from the target DNA regions (22); i.e., an overall transcriptional adaptation process is needed to start growing on Fru. The shorter lag phase of the Δ*cra* strain is to be expected since such transcriptional adaptation (and the ensuing derepression of the genes encoding PTS^Fru^) is not relevant in this genetic background. As expected, this reduced lag phase in cultures of the Δ*cra* mutant was observed only on Fru, while when using the other carbon sources (either Glu or Suc), where the regulation exerted by Cra^*P. putida*^ is not significant for their utilization, no differences were observed. On the other hand, the most important physiological effect of deleting *cra* could be observed in the specific growth rate. We propose that the absence of the Cra^*P. putida*^ transcriptional regulator not only increases the expression of the components of the PTS^Fru^ but also boosts the phosphate flux from GA3P to PEP due to the complete derepression of *PP_3443*. Once again, this effect on the specific growth rate is not observed in Glu or Suc as the carbon source (where it is not needed).

Quantitative physiology experiments with the Δ*PP_3443* mutant of *P. putida* KT2440 were also conducted to check the impact of the cognate GAPDH activity on the overall cell physiology. Under all the tested conditions, the absence of the *PP_3443* gene resulted in a longer lag phase and a growth rate lower than that of the wild-type strain (Fig. 7). Although there are other genes encoding GAPDH activities in the genome of strain KT2440, PP_3443 seems to be relevant under both glycolytic and gluconeogenic growth conditions, a finding in line with the role observed for this enzyme in glycerol cultures (35). However, the most prominent effect was observed when Fru was used as the carbon source. Under such conditions, the Δ*PP_3443* mutant had a (i) 75% longer lag phase (Fig. 7A) and (ii) 32% lower specific growth rate (Fig. 7B) with respect to the wild-type strain. These results point, again, to a relevant role of the GAPDH activity encoded by *PP_3443* in the catabolism of Fru.

### Conclusion.

**FIG 8:**
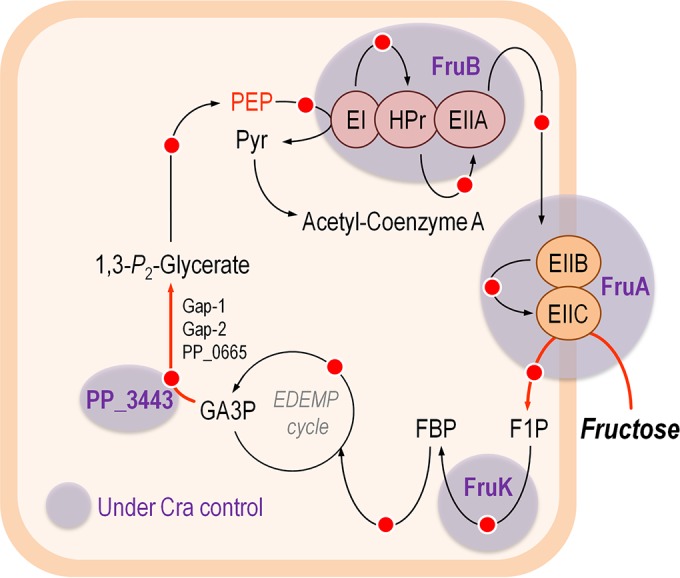
Regulatory duties of Cra in *Pseudomonas putida* KT2440 and metabolic widget for fructose uptake. The diagram sketches the functional relationship between the relevant elements that rule fructose uptake in *P. putida* KT2440. The transcriptional regulator Cra modulates the expression of the *fruBKA* operon, encoding the enzymes needed for fructose transport and phosphorylation, and it also controls the glyceraldehyde-3-P dehydrogenase activity through the activation of *PP_3443* to ensure a high-enough availability of high-energy phosphate (indicated in this outline by a red dot) needed to fulfill the fructose phosphorylation step using phosphoenolpyruvate (PEP) as the phosphate donor. Note that in strain KT2440, the operation of central carbon metabolism is organized around the EDEMP cycle, which encompasses enzymes from the Embden-Meyerhof-Parnas, Entner-Doudoroff, and pentose phosphate pathways (11). GA3P, glyceraldehyde-3-P; FBP, fructose-1,6-P_2_; F1P, fructose-1-P; Pyr, pyruvate.

The results presented in this work revealed two important findings: (i) unlike the case in *E. coli*, Cra^*P. putida*^ is alien to the regulation of many genes related to CCM, and (ii) Cra^*P. putida*^ regulates transcriptionally the expression of the *PP_3443* gene, thereby affecting the overall GAPDH activity of strain KT2440 and the intracellular availability of PEP. The regulation of Cra^*P. putida*^ on *PP_3443* is further wired to the activity of PTS^Fru^ in this bacterium, since in cultures using Fru as the sole carbon source, Cra^*P. putida*^ derepresses the expression of *PP_3443* to increase the total GAPDH activity and therefore ensures sufficient PEP levels for Fru uptake through PTS^Fru^. A model of this remarkable metabolic device, which we termed a metabolic widget, is presented in Fig. 8. The metabolic setting where Cra^*P. putida*^ exerts its regulation is key for the cell: the GA3P node, a hub of CCM. At this point, carbon and phosphate fluxes can be adjusted according to nutritional requirements and/or environmental cues. Cra^*P. putida*^ orchestrates the operation of PTS^Fru^ in a more precise fashion than previously thought: this is because the regulator not only controls the expression of components of the transport system (i.e., the *fruBKA* operon) but also regulates the phosphate flux in CCM with a sort of memory patch. This situation ensures the availability of enough of the PEP needed for the phosphorylation of the PTS components all the way up to exogenous Fru during shifts between conditions characterized by different levels of hexose availability. This situation is reminiscent of the regulatory phenomenon observed when *P. putida* cells grow on glycerol as the carbon source (39). Under these conditions, this bacterium displays a sort of memory, ruled by the GlpR regulator, that enables the cells to better adapt to environmental situations in which glycerol is highly abundant. The regulatory scope of the Cra transcriptional factor in *P. putida* KT2440 seems to be mostly related to the control of Fru uptake, while the corresponding *E. coli* orthologue seems to have divergently evolved to regulate the expression of several CCM genes in response to the internal glycolytic flux. The work above thus exemplifies how the same biochemical and regulatory constituents of a functionality shared by different bacteria might evolve quite differently depending on subtle changes in the connectivity of the cognate molecular ingredients.

## MATERIALS AND METHODS

### Bacterial strains, culture media, and growth conditions.

The reference strain *P. putida* KT2440 and its *Δcra* and *ΔPP_3443* derivatives have been described previously (12, 20, 35). All *P. putida* strains were aerobically grown batchwise at 30°C with shaking at 170 rpm on LB medium or M9 minimal medium (40) containing either Glu, Fru, or Suc at 10 mM as the sole carbon and energy source. Growth was estimated by measuring the optical density at 600 nm (OD_600_), assessed in an Ultrospec 3000 pro UV/visible spectrophotometer (GE Healthcare Bio-Sciences Corp., Piscataway, NJ). For determination of growth parameters, cell cultures were harvested by centrifugation (15 min, 4,000 × *g*, 4°C), washed twice with 10 mM MgSO_4_, and finally resuspended in 10 mM MgSO_4_ to an OD_600_ of ~3. Culture media with the appropriate carbon source were inoculated to an OD_600_ of 0.05 from this cell suspension. Finally, 200-μl aliquots of the freshly inoculated suspension were distributed in microtiter plates (clear-bottom polypropylene 96-well plates; Nunc A/S, Roskilde, Denmark) and were incubated at 30°C with 2 min of orbital shaking every 15 min (41, 42). Bacterial growth was estimated by periodically monitoring the OD_600_ in a SpectraMax Plus384 microplate reader (Molecular Devices, Sunnyvale, CA). Kinetic growth parameters, including the duration of the lag phase and the specific growth rate, were calculated as described elsewhere (43–46).

### RNA manipulation and deep sequencing of transcripts.

Total RNA was extracted by using the RNeasy kit (Qiagen, Valencia, CA), and RNase-free DNase (Qiagen) treatment was performed during the isolation procedure to eliminate any residual DNA in the preparation. The quality of RNA samples was evaluated in an Agilent 2100 Bioanalyzer (Agilent Technologies, Santa Clara, CA). RNA library construction and sequencing were carried out by BGI (Shenzhen, China), using the Illumina mRNA sequencing sample preparation kit (catalog no. RS-930-1001) and the Illumina HiSeq 2000 system (Illumina, Inc., San Diego, CA). Reads generated by the sequencing machines were cleaned and mapped to the database of *Pseudomonas* gene sequences (NCBI reference sequence NC_002947, version NC_002947.3) (47) using SOAP2 software (version 2.21) (48), and the resulting alignment was visualized using IGV software (49). Fold changes and *P* values were calculated as described by Audic and Claverie (50). As the RNA sequencing data generated corresponded to a single biological sample for each carbon source, *P* values were corrected and expressed as false discovery rate values (51, 52). Genes with false discovery rates of ≤0.001 and absolute fold changes larger than 2 were considered differentially expressed. The detailed procedures and the complete set of raw data generated in these experiments are available in a separate study (34).

### Identification of a *cra* operator in the promoter region of *PP_3443*.

The reference *E. coli* consensus sequence for Cra binding sites was retrieved from the PRODORIC database (53, 54). The resulting DNA motif was then searched in the genome of *P. putida* KT2440 by means of the Virtual Footprint software (version 3.0) (55), which revealed the presence of (at least) 89 possible Cra binding sites in the target chromosome, including the site in the promoter region of *PP_3443*.

### Preparation of cell extracts and *in vitro* enzymatic assays.

All the enzyme activity determinations were carried out in cultures during mid-exponential phase (i.e., corresponding to an OD_600_ of ca. 0.5). Cell extracts were prepared starting from 50 ml of culture broth, which was centrifuged at 4,000 × *g* for 10 min. All the following procedures were carried out at 4°C. Cells were washed in 150 mM NaCl and subsequently in 100 mM phosphate-buffered saline (PBS; pH 7.4). The washed pellets were suspended in 500 μl of 100 mM PBS (pH 7.4), supplemented with 1.5 mM 2-mercaptoethanol, and disrupted by ultrasonic treatment (10 treatments of 15 s with 30-s pauses between each round). The mixture was centrifuged at 12,000 × *g* for 5 min, and the supernatant was collected and kept on ice. Enzyme activities were normalized by determining the total cell protein concentration using a Bradford-based (56, 57) protein assay purchased from Sigma-Aldrich Co. We used an extinction coefficient (ε_NADH_) of 6.22 mM^−1^ cm^−1^, representing the difference between the extinction coefficients of NAD(P)H and NAD(P)^+^. One unit of enzyme activity was defined as the quantity of enzyme that catalyzed the formation of 1 μmol of the corresponding product during the time indicated and at 30°C. GAPDH activity was measured by using the protocol described by Tiwari and Campbell (58) as modified by Nikel et al. (35). The assay mixture contained (in a final volume of 1.0 ml) 100 mM Tris-HCl (pH 7.5), 6 mM d,l-GA3P, 10 mM cysteine-HCl, 15 mM NaH_2_AsO_4_, 20 mM NaF, 0.35 mM NAD^+^ or NADP^+^, and 50 to 150 μl of the cell extract. The GA3P and cysteine-HCl solutions were neutralized just before use. The reaction mixture without GA3P was incubated at 30°C for 1 min, and the reaction was initiated by adding the substrate. The reduction of either NAD^+^ or NADP^+^ was followed by taking readings every 10 s for 15 min at 340 nm.

### *In silico* modeling and simulations.

The kinetic model that represents the reactions (see Fig. S4A in the supplemental material) of the system at stake was built deterministically with a set of ODEs (see Fig. S4B in the supplemental material). Due to the high number of connections modeled that have unknown dynamics, stochastic approaches are to be considered noninformative for the time being. Simulations were carried out using our own in-house program coded in Python as the programming language. Each of the plots in Fig. 5 and 6 shows a number of simulations, in particular, one per graph coordinate at intervals of 0.1 along the *x* axis. All the simulations began with the system at a pristine initial state; thus, the concentration of the output molecular species was set at 0 μM. The measurements plotted correspond to the values of the system at a steady state after 0.5 h of evolution. All the graphs are represented in a logarithmic scale in both axes to facilitate the inspection of the system at low *k*_17_ values, where the dynamics revealed a very specific behavior as described in Results and Discussion.

### Metabolic flux analysis.

Data for the distribution of metabolic fluxes in *P. putida* KT2440 growing on different substrates were taken from our previous publications (11, 12) and analyzed as described by Nikel and Chavarría (36).

### Accession number(s).

The complete raw data set of the deep RNA sequencing analysis is publicly available at the Gene Expression Omnibus site of the NCBI (http://www.ncbi.nlm.nih.gov/geo) with GenBank accession number GSE46491.

## References

[1] del Castillo T, Ramos JL, Rodríguez-Herva JJ, Fuhrer T, Sauer U, Duque E 2007 Convergent peripheral pathways catalyze initial glucose catabolism in *Pseudomonas putida*: genomic and flux analysis. J Bacteriol 189:5142–5152. doi:10.1128/JB.00203-07. 17483213 PMC1951859

[2] Jahreis K, Pimentel-Schmitt EF, Brückner R, Titgemeyer F 2008 Ins and outs of glucose transport systems in eubacteria. FEMS Microbiol Rev 32:891–907. doi:10.1111/j.1574-6976.2008.00125.x. 18647176

[3] Iyer B, Rajput MS, Jog R, Joshi E, Bharwad K, Rajkumar S 2016 Organic acid mediated repression of sugar utilization in rhizobia. Microbiol Res 192:211–220. doi:10.1016/j.micres.2016.07.006. 27664739

[4] Pflüger-Grau K, de Lorenzo V 2014 From the phosphoenolpyruvate phosphotransferase system to selfish metabolism: a story retraced in *Pseudomonas putida*. FEMS Microbiol Lett 356:144–153. doi:10.1111/1574-6968.12459. 24801646

[5] Clarke PH 1982 The metabolic versatility of pseudomonads. Antonie van Leeuwenhoek 48:105–130. doi:10.1007/BF00405197. 6808915

[6] Somavanshi R, Ghosh B, Sourjik V 2016 Sugar influx sensing by the phosphotransferase system of *Escherichia coli*. PLoS Biol 14:e2000074. doi:10.1371/journal.pbio.2000074. 27557415 PMC4996493

[7] Luo Y, Zhang T, Wu H 2014 The transport and mediation mechanisms of the common sugars in *Escherichia coli*. Biotechnol Adv 32:905–919. doi:10.1016/j.biotechadv.2014.04.009. 24780155

[8] Deutscher J, Francke C, Postma PW 2006 How phosphotransferase system-related protein phosphorylation regulates carbohydrate metabolism in bacteria. Microbiol Mol Biol Rev 70:939–1031. doi:10.1128/MMBR.00024-06. 17158705 PMC1698508

[9] Bettenbrock K, Fischer S, Kremling A, Jahreis K, Sauter T, Gilles ED 2006 A quantitative approach to catabolite repression in *Escherichia coli*. J Biol Chem 281:2578–2584. doi:10.1074/jbc.M508090200. 16263707

[10] Nikel PI, Chavarría M, Danchin A, de Lorenzo V 2016 From dirt to industrial applications: *Pseudomonas putida* as a synthetic biology *chassis* for hosting harsh biochemical reactions. Curr Opin Chem Biol 34:20–29. doi:10.1016/j.cbpa.2016.05.011. 27239751

[11] Nikel PI, Chavarría M, Fuhrer T, Sauer U, de Lorenzo V 2015 *Pseudomonas putida* KT2440 strain metabolizes glucose through a cycle formed by enzymes of the Entner-Doudoroff, Embden-Meyerhof-Parnas, and pentose phosphate pathways. J Biol Chem 290:25920–25932. doi:10.1074/jbc.M115.687749. 26350459 PMC4646247

[12] Chavarría M, Kleijn RJ, Sauer U, Pflüger-Grau K, de Lorenzo V 2012 Regulatory tasks of the phosphoenolpyruvate-phosphotransferase system of *Pseudomonas putida* in central carbon metabolism. mBio 3:e00028-12. doi:10.1128/mBio.00028-12. 22434849 PMC3312210

[13] Nikel PI, Martínez-García E, de Lorenzo V 2014 Biotechnological domestication of pseudomonads using synthetic biology. Nat Rev Microbiol 12:368–379. doi:10.1038/nrmicro3253. 24736795

[14] Kornberg HL 2001 Routes for fructose utilization by *Escherichia coli*. J Mol Microbiol Biotechnol 3:355–359. 11361065

[15] Shimizu K 2016 Metabolic regulation and coordination of the metabolism in bacteria in response to a variety of growth conditions. Adv Biochem Eng Biotechnol 155:1–54. doi:10.1007/10_2015_320. 25712586

[16] Kochanowski K, Volkmer B, Gerosa L, Haverkorn van Rijsewijk BR, Schmidt A, Heinemann M 2013 Functioning of a metabolic flux sensor in *Escherichia coli*. Proc Natl Acad Sci USA 110:1130–1135. doi:10.1073/pnas.1202582110. 23277571 PMC3549114

[17] Shimada T, Yamamoto K, Ishihama A 2011 Novel members of the Cra regulon involved in carbon metabolism in *Escherichia coli*. J Bacteriol 193:649–659. doi:10.1128/JB.01214-10. 21115656 PMC3021228

[18] Keseler IM, Collado-Vides J, Santos-Zavaleta A, Peralta-Gil M, Gama-Castro S, Muñiz-Rascado L, Bonavides-Martinez C, Paley S, Krummenacker M, Altman T, Kaipa P, Spaulding A, Pacheco J, Latendresse M, Fulcher C, Sarker M, Shearer AG, Mackie A, Paulsen I, Gunsalus RP, Karp PD 2011 EcoCyc: a comprehensive database of *Escherichia coli* biology. Nucleic Acids Res 39:D583–D590. doi:10.1093/nar/gkq1143. 21097882 PMC3013716

[19] Belda E, van Heck RGA, López-Sánchez MJ, Cruveiller S, Barbe V, Fraser C, Klenk HP, Petersen J, Morgat A, Nikel PI, Vallenet D, Rouy Z, Sekowska A, Martins dos Santos VAP, de Lorenzo V, Danchin A, Médigue C 2016 The revisited genome of *Pseudomonas putida* KT2440 enlightens its value as a robust metabolic *chassis*. Environ Microbiol 18:3403–3424. doi:10.1111/1462-2920.13230. 26913973

[20] Nelson KE, Weinel C, Paulsen IT, Dodson RJ, Hilbert H, Martins dos Santos VAP, Fouts DE, Gill SR, Pop M, Holmes M, Brinkac L, Beanan M, DeBoy RT, Daugherty S, Kolonay J, Madupu R, Nelson W, White O, Peterson J, Khouri H, Hance I, Chris Lee P, Holtzapple E, Scanlan D, Tran K, Moazzez A, Utterback T, Rizzo M, Lee K, Kosack D, Moestl D, Wedler H, Lauber J, Stjepandic D, Hoheisel J, Straetz M, Heim S, Kiewitz C, Eisen JA, Timmis KN, Düsterhöft A, Tümmler B, Fraser CM 2002 Complete genome sequence and comparative analysis of the metabolically versatile *Pseudomonas putida* KT2440. Environ Microbiol 4:799–808. doi:10.1046/j.1462-2920.2002.00366.x. 12534463

[21] Chavarría M, Durante-Rodríguez G, Krell T, Santiago C, Brezovsky J, Damborsky J, de Lorenzo V 2014 Fructose 1-phosphate is the one and only physiological effector of the Cra (FruR) regulator of *Pseudomonas putida*. FEBS Open Bio 4:377–386. doi:10.1016/j.fob.2014.03.013. PMC4050194 24918052

[22] Chavarría M, Santiago C, Platero R, Krell T, Casasnovas JM, de Lorenzo V 2011 Fructose 1-phosphate is the preferred effector of the metabolic regulator Cra of *Pseudomonas putida*. J Biol Chem 286:9351–9359. doi:10.1074/jbc.M110.187583. 21239488 PMC3058975

[23] Shimada T, Fujita N, Maeda M, Ishihama A 2005 Systematic search for the Cra-binding promoters using genomic SELEX system. Genes Cells 10:907–918. doi:10.1111/j.1365-2443.2005.00888.x. 16115199

[24] Chubukov V, Gerosa L, Kochanowski K, Sauer U 2014 Coordination of microbial metabolism. Nat Rev Microbiol 12:327–340. doi:10.1038/nrmicro3238. 24658329

[25] Ramseier TM 1996 Cra and the control of carbon flux *via* metabolic pathways. Res Microbiol 147:489–493. doi:10.1016/0923-2508(96)84003-4. 9084760

[26] Zhang Z, Aboulwafa M, Saier MH 2014 Regulation of *crp* gene expression by the catabolite repressor/activator, Cra, in *Escherichia coli*. J Mol Microbiol Biotechnol 24:135–141. doi:10.1159/000362722. 24923415 PMC4125524

[27] Yao R, Hirose Y, Sarkar D, Nakahigashi K, Ye Q, Shimizu K 2011 Catabolic regulation analysis of *Escherichia coli* and its *crp*, *mlc*, *mgsA*, *pgi* and *ptsG* mutants. Microb Cell Fact 10:67. doi:10.1186/1475-2859-10-67. 21831320 PMC3169459

[28] Lemuth K, Hardiman T, Winter S, Pfeiffer D, Keller MA, Lange S, Reuss M, Schmid RD, Siemann-Herzberg M 2008 Global transcription and metabolic flux analysis of *Escherichia coli* in glucose-limited fed-batch cultivations. Appl Environ Microbiol 74:7002–7015. doi:10.1128/AEM.01327-08. 18806003 PMC2583496

[29] Sarkar D, Siddiquee KA, Araúzo-Bravo MJ, Oba T, Shimizu K 2008 Effect of *cra* gene knockout together with *edd* and *iclR* genes knockout on the metabolism in *Escherichia coli*. Arch Microbiol 190:559–571. doi:10.1007/s00203-008-0406-2. 18648770

[30] Nanchen A, Schicker A, Revelles O, Sauer U 2008 Cyclic AMP-dependent catabolite repression is the dominant control mechanism of metabolic fluxes under glucose limitation in *Escherichia coli*. J Bacteriol 190:2323–2330. doi:10.1128/JB.01353-07. 18223071 PMC2293195

[31] Perrenoud A, Sauer U 2005 Impact of global transcriptional regulation by ArcA, ArcB, Cra, Crp, Cya, Fnr, and Mlc on glucose catabolism in *Escherichia coli*. J Bacteriol 187:3171–3179. doi:10.1128/JB.187.9.3171-3179.2005. 15838044 PMC1082841

[32] Ramseier TM, Bledig S, Michotey V, Feghali R, Saier MH 1995 The global regulatory protein FruR modulates the direction of carbon flow in *Escherichia coli*. Mol Microbiol 16:1157–1169. doi:10.1111/j.1365-2958.1995.tb02339.x. 8577250

[33] van Vliet AH 2010 Next generation sequencing of microbial transcriptomes: challenges and opportunities. FEMS Microbiol Lett 302:1–7. doi:10.1111/j.1574-6968.2009.01767.x. 19735299

[34] Kim J, Oliveros JC, Nikel PI, de Lorenzo V, Silva-Rocha R 2013 Transcriptomic fingerprinting of *Pseudomonas putida* under alternative physiological regimes. Environ Microbiol Rep 5:883–891. doi:10.1111/1758-2229.12090. 24249296

[35] Nikel PI, Kim J, de Lorenzo V 2014 Metabolic and regulatory rearrangements underlying glycerol metabolism in *Pseudomonas putida* KT2440. Environ Microbiol 16:239–254. doi:10.1111/1462-2920.12224. 23967821

[36] Nikel PI, Chavarría M 2016 Quantitative physiology approaches to understand and optimize reducing power availability in environmental bacteria, p 39–70. *In* McGenity TJ, Timmis KN, Nogales-Fernández B (ed), Hydrocarbon and lipid microbiology protocols. Synthetic and systems biology. Tools. Humana Press, Heidelberg, Germany. doi:10.1007/8623_2015_84.

[37] Clore GM, Venditti V 2013 Structure, dynamics and biophysics of the cytoplasmic protein-protein complexes of the bacterial phosphoenolpyruvate:sugar phosphotransferase system. Trends Biochem Sci 38:515–530. doi:10.1016/j.tibs.2013.08.003. 24055245 PMC3831880

[38] Noor E, Eden E, Milo R, Alon U 2010 Central carbon metabolism as a minimal biochemical walk between precursors for biomass and energy. Mol Cell 39:809–820. doi:10.1016/j.molcel.2010.08.031. 20832731

[39] Nikel PI, Romero-Campero FJ, Zeidman JA, Goñi-Moreno Á, de Lorenzo V 2015 The glycerol-dependent metabolic persistence of *Pseudomonas putida* KT2440 reflects the regulatory logic of the GlpR repressor. mBio 6:e00340-15. doi:10.1128/mBio.00340-15. 25827416 PMC4453509

[40] Green MR, Sambrook J 2012 Molecular cloning: a laboratory manual, 4th ed. Cold Spring Harbor Laboratory Press, Cold Spring Harbor, NY.

[41] Martínez-García E, Nikel PI, Aparicio T, de Lorenzo V 2014 *Pseudomonas* 2.0: genetic upgrading of *P. putida* KT2440 as an enhanced host for heterologous gene expression. Microb Cell Fact 13:159. doi:10.1186/s12934-014-0159-3. 25384394 PMC4230525

[42] Martínez-García E, Aparicio T, de Lorenzo V, Nikel PI 2014 New transposon tools tailored for metabolic engineering of Gram-negative microbial cell factories. Front Bioeng Biotechnol 2:46. doi:10.3389/fbioe.2014.00046. 25389526 PMC4211546

[43] Dalgaard P, Koutsoumanis K 2001 Comparison of maximum specific growth rates and lag times estimated from absorbance and viable count data by different mathematical models. J Microbiol Methods 43:183–196. doi:10.1016/S0167-7012(00)00219-0. 11118653

[44] Nikel PI, de Lorenzo V 2013 Engineering an anaerobic metabolic regime in *Pseudomonas putida* KT2440 for the anoxic biodegradation of 1,3-dichloroprop-1-ene. Metab Eng 15:98–112. doi:10.1016/j.ymben.2012.09.006. 23149123

[45] Nikel PI, de Lorenzo V 2013 Implantation of unmarked regulatory and metabolic modules in Gram-negative bacteria with specialised mini-transposon delivery vectors. J Biotechnol 163:143–154. doi:10.1016/j.jbiotec.2012.05.002. 22609234

[46] Nikel PI, Pérez-Pantoja D, de Lorenzo V 2013 Why are chlorinated pollutants so difficult to degrade aerobically? Redox stress limits 1,3-dichloroprop-1-ene metabolism by *Pseudomonas* *pavonaceae*. Philos Trans R Soc Lond B Biol Sci 368:20120377. doi:10.1098/rstb.2012.0377. PMC3638467 23479756

[47] Winsor GL, Griffiths EJ, Lo R, Dhillon BK, Shay JA, Brinkman FS 2016 Enhanced annotations and features for comparing thousands of *Pseudomonas* genomes in the *Pseudomonas* genome database. Nucleic Acids Res 44:D646–D653. doi:10.1093/nar/gkv1227. 26578582 PMC4702867

[48] Li R, Yu C, Li Y, Lam TW, Yiu SM, Kristiansen K, Wang J 2009 SOAP2: an improved ultrafast tool for short read alignment. Bioinformatics 25:1966–1967. doi:10.1093/bioinformatics/btp336. 19497933

[49] Thorvaldsdóttir H, Robinson JT, Mesirov JP 2013 Integrative genomics viewer (IGV): high-performance genomics data visualization and exploration. Brief Bioinform 14:178–192. doi:10.1093/bib/bbs017. 22517427 PMC3603213

[50] Audic S, Claverie JM 1997 The significance of digital gene expression profiles. Genome Res 7:986–995. doi:10.1101/gr.7.10.986. 9331369

[51] Benjamini Y, Drai D, Elmer G, Kafkafi N, Golani I 2001 Controlling the false discovery rate in behavior genetics research. Behav Brain Res 125:279–284. doi:10.1016/S0166-4328(01)00297-2. 11682119

[52] Lippolis JD, Brunelle BW, Reinhardt TA, Sacco RE, Thacker TC, Looft TP, Casey TA 2016 Differential gene expression of three mastitis-causing *Escherichia coli* strains grown under planktonic, swimming, and swarming culture conditions. mSystems 1:e00064-16. doi:10.1128/mSystems.00064-16. 27822550 PMC5072449

[53] Grote A, Klein J, Retter I, Haddad I, Behling S, Bunk B, Biegler I, Yarmolinetz S, Jahn D, Münch R 2009 PRODORIC (release 2009): a database and tool platform for the analysis of gene regulation in prokaryotes. Nucleic Acids Res 37:D61–D65. doi:10.1093/nar/gkn837. 18974177 PMC2686542

[54] Münch R, Hiller K, Barg H, Heldt D, Linz S, Wingender E, Jahn D 2003 PRODORIC: prokaryotic database of gene regulation. Nucleic Acids Res 31:266–269. doi:10.1093/nar/gkg037. 12519998 PMC165484

[55] Münch R, Hiller K, Grote A, Scheer M, Klein J, Schobert M, Jahn D 2005 Virtual footprint and PRODORIC: an integrative framework for regulon prediction in prokaryotes. Bioinformatics 21:4187–4189. doi:10.1093/bioinformatics/bti635. 16109747

[56] Bradford MM 1976 A rapid and sensitive method for the quantitation of microgram quantities of protein utilizing the principle of protein-dye binding. Anal Biochem 72:248–254. doi:10.1016/0003-2697(76)90527-3. 942051

[57] Ruiz JA, Fernández RO, Nikel PI, Méndez BS, Pettinari MJ 2006 Dye (*arc*) mutants: insights into an unexplained phenotype and its suppression by the synthesis of poly(3-hydroxybutyrate) in *Escherichia coli* recombinants. FEMS Microbiol Lett 258:55–60. doi:10.1111/j.1574-6968.2006.00196.x. 16630255

[58] Tiwari NP, Campbell JJ 1969 Enzymatic control of the metabolic activity of *Pseudomonas aeruginosa* grown in glucose or succinate media. Biochim Biophys Acta 192:395–401. doi:10.1016/0304-4165(69)90388-2. 4312775

[59] Dublanche Y, Michalodimitrakis K, Kümmerer N, Foglierini M, Serrano L 2006 Noise in transcription negative feedback loops: simulation and experimental analysis. Mol Syst Biol 2:41. doi:10.1038/msb4100081. 16883354 PMC1681513

[60] Miró-Bueno JM, Rodríguez-Patón A 2011 A simple negative interaction in the positive transcriptional feedback of a single gene is sufficient to produce reliable oscillations. PLoS One 6:e27414. doi:10.1371/journal.pone.0027414. 22205920 PMC3244268

