# Supplementary material for: A Metabolic Widget Adjusts the Phosphoenolpyruvate-Dependent Fructose Influx in Pseudomonas putida
Source: mSystems. 2016 Dec 6;1(6):e00154-16. doi: 10.1128/mSystems.00154-16 (PMC5141268; doi:10.1128/mSystems.00154-16)
Supplement: Figure S1 [file sys006162068sf1.pdf]

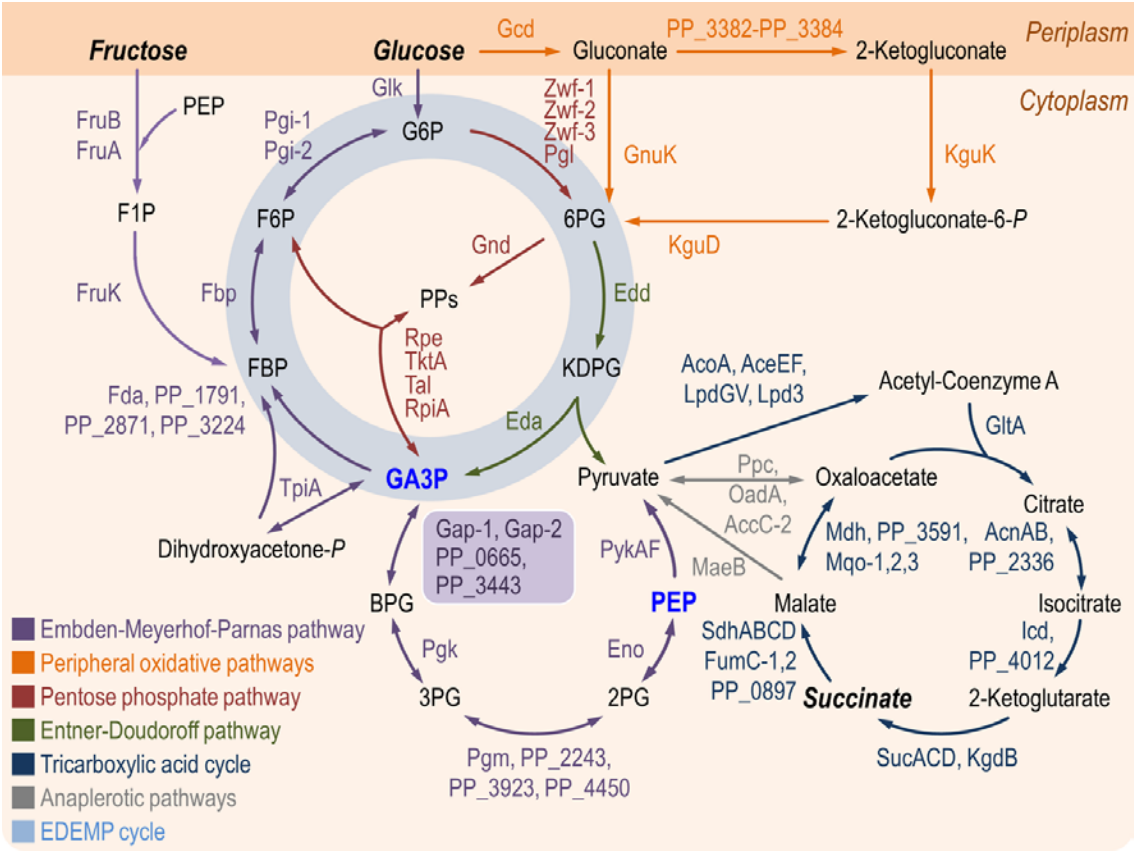

Fig. S1. Central carbon metabolism in *Pseudomonas putida* KT2440. Representation of glucose, fructose, and succinate metabolism in *P. putida* KT2440. Individual components of different metabolic blocks within central carbon metabolism are highlighted with different colors as indicated in the lower corner to the left. The abbreviations for the metabolites and enzymes in this metabolic map are as follows: Glk, glucokinase; G6P, glucose-6-*P*; 6PG, 6-phosphogluconate; KDPG, 2-keto-3-deoxy-6-phosphogluconate; GnuK, gluconokinase; Gcd, glucose dehydrogenase; FBP, fructose-1,6-P_2; Fbp, fructose-1,6-bisphosphatase; Pgi, G6P isomerase; Zwf, G6P dehydrogenase; Pgl, 6-phosphogluconolactonase; KguK, 2-ketogluconate kinase; KguD, 2-ketogluconate-6-*P* reductase; Edd, 6PG dehydratase; Eda, KDPG aldolase; Fda, FBP aldolase; TpiA, triose phosphate isomerase; Gnd, 6PG dehydrogenase; PPs, pool of pentoses phosphate; Rpe, ribulose-5-*P* 3-epimerase; TktA, transketolase; Tal, transaldolase; RpiA, ribose-5-*P* isomerase; GA3P, glyceraldehyde-3-*P*; BPG, 1,3-P_2-Glycerate; 3PG, glycerate-3-*P*; 2PG, glycerate-2-*P*; Pgk, phosphoglycerate kinase; Pgm, phosphoglycerate mutase; Eno, phosphopyruvate hydratase; PEP, phosphoenolpyruvate; AceEF, LpdGV, Lpd3, and AcoA, pyruvate dehydrogenase; MaeB, malic enzyme; Pyk, pyruvate kinase; Mdh, malate dehydrogenase; Mqo, malate:quinone oxidoreductase; AccC-2, pyruvate carboxylase subunit A; OadA, pyruvate carboxylase subunit B; Ppc, PEP carboxylase; FumC, fumarate hydratase; SdhABCD, succinate dehydrogenase; SucACD and KgdB, succinyl-coenzyme A synthetase and 2-ketoglutarate dehydrogenase; AcnAB, aconitate hydratase; Icd, isocitrate dehydrogenase; GltA, citrate synthase; FruB/FruA, PTS^{Fru} system; and FruK, 1-phosphofructokinase. Note that the metabolic node connecting GA3P with downwards metabolism is indicated by highlighting the four GA3P dehydrogenase enzymes of strain KT2440. The two key metabolites involved in the *metabolic widget* described in this work (i.e., GA3P and PEP) are indicated in blue.
