# Supplementary material for: A Metabolic Widget Adjusts the Phosphoenolpyruvate-Dependent Fructose Influx in Pseudomonas putida
Source: mSystems. 2016 Dec 6;1(6):e00154-16. doi: 10.1128/mSystems.00154-16 (PMC5141268; doi:10.1128/mSystems.00154-16)
Supplement: Figure S2 [file sys006162068sf2.pdf]

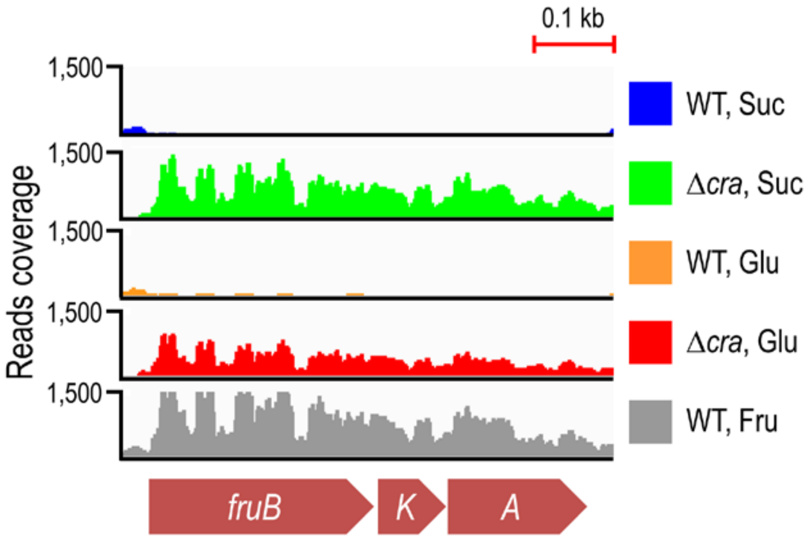

Fig. S2. Sequence coverage plots for the *fruBKA* gene cluster of *Pseudomonas putida* KT2440 and its Δ*cra* derivative analyzed at the single-nucleotide level. Data correspond to the expression pattern for samples taken during the mid-logarithmic phase of growth in cultures developed on glucose (Glu), fructose (Fru), or succinate (Suc) as the sole carbon source. WT, wild-type strain.
