# Supplementary material for: A Metabolic Widget Adjusts the Phosphoenolpyruvate-Dependent Fructose Influx in Pseudomonas putida
Source: mSystems. 2016 Dec 6;1(6):e00154-16. doi: 10.1128/mSystems.00154-16 (PMC5141268; doi:10.1128/mSystems.00154-16)
Supplement: Table S1 [file sys006162068st6.pdf]

Table S1. Hypothetical DNA binding sites of the Cra protein in the chromosome of *Pseudomonas putida* KT2440 obtained with the PRODORIC database[a].

Start	End	Sequence (5' →3')	Gene or locus	Product name	ATG distance	Location
103256	103267	TGAAGCGCTTCT	*PP_0098*	Hypothetical protein	261	C
351298	351309	TGAAGCGTTTTC	*hisA (PP_0292)*	Phosphoribosyl formimino-5-aminoimidazole carboxamide ribotideisomerase	204	C
360174	360185	TGAATGGCTTCA	*PP_0300*	Metallopeptidase	163	C
650208	650219	TGAAACGTTTCT	*accB (PP_0559)*	Acetyl-CoA carboxylase biotin carboxyl carrier protein subunit	54	I
826630	826641	TGAAGCGATTCT	*PP_0711*	Isochorismatase superfamily hydrolase	167	C
909364	909375	TGAAACGTTTAA	*cra (PP_0792)*	DNA-binding transcriptional regulator Cra	158	I
909364	909375	TGAAACGTTTAA	*fruB (PP_0793)*	Phosphoenolpyruvate-protein phosphotransferase	116	I
1033039	1033050	TGAAGCGGTTAT	*PP_0891*	Hypothetical protein	329	C
1151061	1151072	TGAAACGGTTTT	*gap-1 (PP_1009)*	Glyceraldehyde-3-*P* dehydrogenase, type I	52	I
1151061	1151072	TGAAACGGTTTT	*edd (PP_1010)*	Phosphogluconate dehydratase	202	I
1355962	1355973	TGAACCGTTTTT	*PP_1181*	Winged helix family, two component transcriptional regulator	150	I
1393535	1393546	TGAAGCGCTTAC	*ruvA (PP_1216)*	Holliday junction DNA helicase RuvA	102	C
1427684	1427695	TGAATCGTTTTT	*mqo-2 (PP_1251)*	Malate:quinone oxidoreductase	203	I
1648807	1648818	TGAAGCGTTTCA	*oprB-2 (PP_1445)*	Porin	90	I
1897251	1897262	TGAATCGTTTTC	*sugE (PP_1701)*	Small multidrug resistance protein	181	I
2090364	2090375	TGAATCGATAAT	*PP_1868*	DEAD/DEAH box helicase	297	I
2344873	2344884	TGAATCGTTTGC	*PP_2061*	Hypothetical protein	227	C
2589715	2589726	TGAATCGCTTCG	*PP_2267*	Phage single-stranded DNA-binding protein	225	I
2772963	2772974	TGAATCGTTTCA	*PP_2425*	AraC family transcriptional regulator	151	I
2772963	2772974	TGAATCGTTTCA	*PP_2426*	D-Isomer specific 2-hydroxyacid dehydrogenase	20	I
2879250	2879261	TGAATCGGTTCC	*PP_2533*	D-Isomer specific 2-hydroxyacid dehydrogenase	248	C
3226859	3226870	TGAATGGTTTTT	*PP_2828*	Hypothetical protein	289	I
3270160	3270171	TGAATCGCTTCT	*PP_2868*	Cro/c*I* family transcriptional regulator	71	C
3899537	3899548	TGAATCGTTTCA	*PP_3443*	Glyceraldehyde-3-*P* dehydrogenase	42	C
4283210	4283221	TGAATCGCTTTC	*PP_3754*	β-Ketothiolase	175	C
4298073	4298084	TGAAACGTTTAT	*PP_3768*	Shikimate 5-dehydrogenase	4	I
4298073	4298084	TGAAACGTTTAT	*PP_3769*	Hypothetical protein	129	I

4310165	4310176	TGAAGCGCTTAT	*PP_3783*	Hypothetical protein	111	C
4932527	4932538	TGAACCGTTTCC	*fliA (PP_4341)*	Flagellar biosynthesis σ factor	311	C
5270454	5270465	TGAAGCGTTTAA	*radA (PP_4644)*	DNA repair protein RadA	118	I
5270454	5270465	TGAAGCGTTTAA	*mscL (PP_4645)*	Large-conductance mechanosensitive channel	48	I
5283473	5283484	TGAAACGCTTTT	*PP_4658*	Methyl-accepting chemotaxis sensory transducer	110	I
5319697	5319708	TGAAGCGATTTT	*ilvB (PP_4680)*	Acetolactate synthase 3 catalytic subunit	326	I
5374325	5374336	TGAAGCGCTTCT	*dapB (PP_4725)*	Dihydrodipicolinate reductase	28	C
5475045	5475056	TGAATGGCTTAA	*proA (PP_4811)*	γ-Glutamyl-*P* reductase	254	I
5475045	5475056	TGAATGGCTTAA	*PP_4812*	DNA glycosylase	41	I
5635367	5635378	TGAAACGTTTCC	*PP_4948*	Acyl-CoA dehydrogenase	250	I
5753600	5753611	TGAAACGTTTAA	*ntrB (PP_5047)*	Signal transduction histidine kinase, nitrogen specific	185	I
5798118	5798129	TGAAGCGTTTCA	*gltB (PP_5076)*	Glutamate synthase subunit α	228	I
6079513	6079524	TGAATCGTTTAC	*PP_5330*	Major facilitator family transporter	297	I
6079513	6079524	TGAATCGTTTAC	*PP_5331*	Thioesterase	54	I
6163277	6163288	TGAATCGATTGA	*PP_5404*	Transposition protein, TnsC-like protein	14	C

[a] Possible binding sites were identified using the PRODORIC database (1, 2). The two first columns of the table represent the coordinates of the putative binding site in nucleotides. In the instances in which no gene name has been assigned to the corresponding open reading frame, the PP number is given according to the annotations of Nelson *et al*. (3) and Belda *et al*. (4). C, coding region; I, intergenic region.

References

1. Grote A, Klein J, Retter I, Haddad I, Behling S, Bunk B, Biegler I, Yarmolinetz S, Jahn D, Münch R. 2009. PRODORIC (release 2009): a database and tool platform for the analysis of gene regulation in prokaryotes. Nucleic Acids Res. 37:D61-D65. http://dx.doi.org/10.1093/nar/gkn837
2. Münch R, Hiller K, Barg H, Heldt D, Linz S, Wingender E, Jahn D. 2003. PRODORIC: prokaryotic database of gene regulation. Nucleic Acids Res. 31:266-269. http://dx.doi.org/10.1093/nar/gkg037
3. Nelson KE, Weinel C, Paulsen IT, Dodson RJ, Hilbert H, Martins dos Santos VAP, Fouts DE, Gill SR, Pop M, Holmes M, Brinkac L, Beanan M, DeBoy RT, Daugherty S, Kolonay J, Madupu R, Nelson W, White O, Peterson J, Khouri H, Hance I, Chris Lee P, Holtzapple E, Scanlan D, Tran K, Moazzez A, Utterback T, Rizzo M, Lee K, Kosack D, Moestl D, Wedler H, Lauber J, Stjepandic D, Hoheisel J, Straetz M, Heim S, Kiewitz C, Eisen JA, Timmis KN, Düsterhöft A, Tümmler B, Fraser CM. 2002. Complete genome sequence and comparative analysis of the metabolically versatile *Pseudomonas putida* KT2440. Environ. Microbiol. 4:799-808. http://dx.doi.org/10.1046/j.1462-2920.2002.00366.x
4. Belda E, van Heck RGA, López-Sánchez MJ, Cruveiller S, Barbe V, Fraser C, Klenk HP, Petersen J, Morgat A, Nikel PI, Vallenet D, Rouy Z, Sekowska A, Martins dos Santos VAP, de Lorenzo V, Danchin A, Médigue C. 2016. The revisited genome of *Pseudomonas putida* KT2440 enlightens its value as a robust metabolic *chassis*. Environ. Microbiol., In press, http://dx.doi.org/10.1111/1462-2920.13230
