# Supplementary material for: A Metabolic Widget Adjusts the Phosphoenolpyruvate-Dependent Fructose Influx in Pseudomonas putida
Source: mSystems. 2016 Dec 6;1(6):e00154-16. doi: 10.1128/mSystems.00154-16 (PMC5141268; doi:10.1128/mSystems.00154-16)
Supplement: Figure S3 [file sys006162068sf3.pdf]

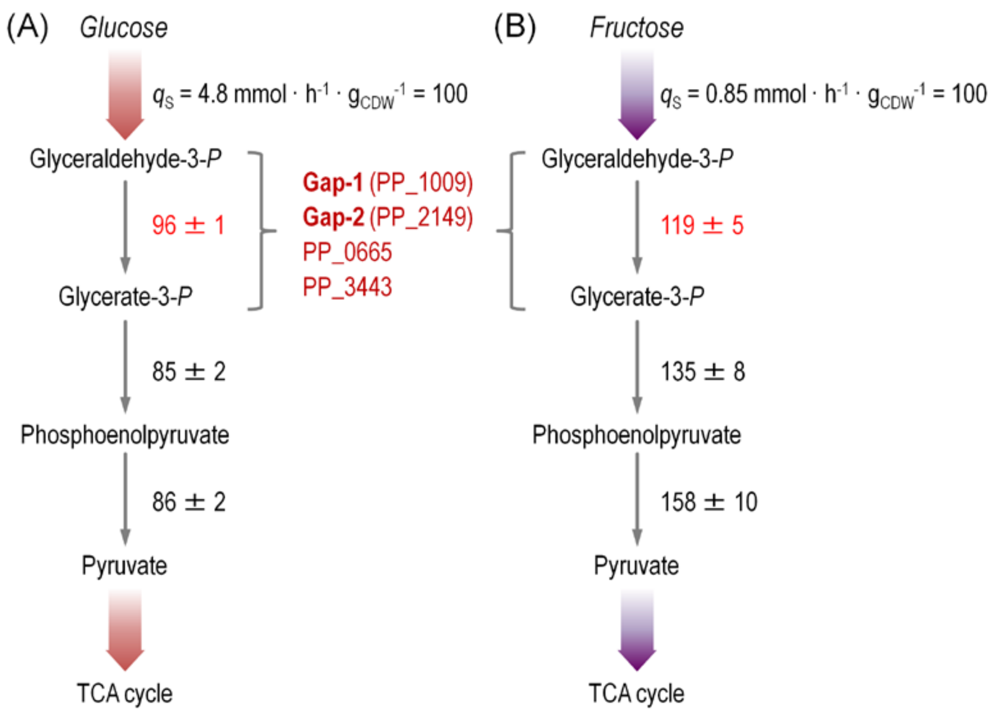

Fig. S3. Net metabolic fluxes around the glyceraldehyde-3-*P* node of wild-type *Pseudomonas putida* grown on glucose and fructose. The value of each metabolic flux in this sub-network was normalized to the specific carbon source uptake rate (arbitrarily set to 100, actual q_S values are also given in the sketch), for wild-type *P*. *putida* growing on either glucose (A) or fructose (B), and only the fluxes connecting glyceraldehyde-3-*P* to pyruvate are shown in this sketch. Note that the reactions connecting these metabolites with the rest of the metabolic network are indicated by means of wide shaded arrows. The reaction catalyzed by glyceraldehyde-3-*P* dehydrogenase is shown in red along the four isozymes responsible for this transformation in strain KT2440. CDW, cell dry weight.
