# Supplementary material for: A Metabolic Widget Adjusts the Phosphoenolpyruvate-Dependent Fructose Influx in Pseudomonas putida
Source: mSystems. 2016 Dec 6;1(6):e00154-16. doi: 10.1128/mSystems.00154-16 (PMC5141268; doi:10.1128/mSystems.00154-16)
Supplement: Figure S4 [file sys006162068sf4.pdf]

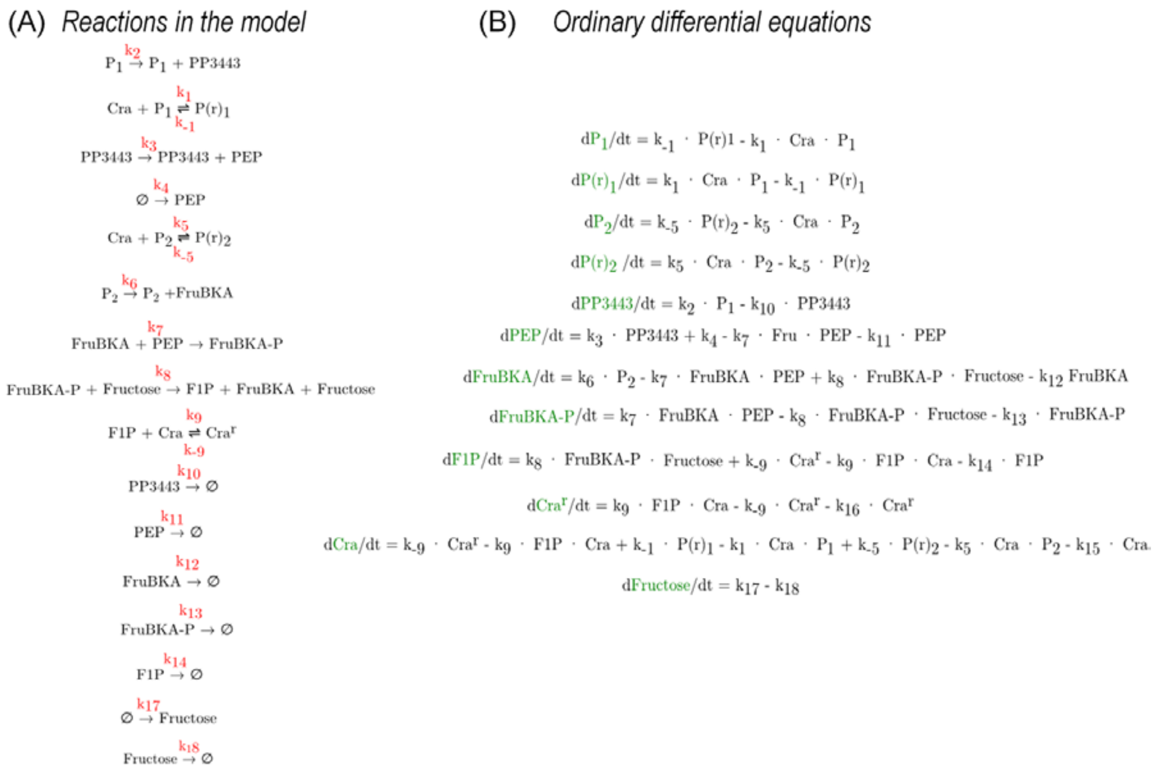

Fig. S4. Reactions used in the kinetic model and ordinary differential equations utilized for the calculations. The kinetic model proposed in Fig. 5 in the main text is composed of a series of reactions (A), that were interpreted by means of a set of ordinary differential equations (B). In the model, k_1 and k_{-1} represent the binding and unbinding rates, respectively, of the Cra regulator to promoter P_1 (controlling the expression of *PP_3443*). They were estimated to have the same value as k_5 and k_{-5}, experimentally measured in a previous work, and they represent the binding and unbinding rates, respectively, of the Cra regulator to promoter P_2 (controlling the expression of *fruBKA*). The k_2 parameter merges transcription and translation into a single event (represented as such for the sake of simplicity). Rates k_3 and k_4 describe the production of phosphoenolpyruvate (PEP) either from PP_3443 or other sources, respectively. These values were adjusted to experimental observations. The k_6 parameter represents the transcription and translation of *fruBKA*. Rates k_7 and k_8 represent 2:1 reactions, the former indicates the formation of FruBKA-P and the later represents the generation of fructose-1-*P* (F1P). Finally, k_9 and k_{-9} are the constants for binding/unbinding of F1P and the Cra regulator. Besides these parameters, two different sets of degradation constants were defined: the first set for *proteins* and the second one for *metabolites*. The rates for the first group (k_{10}, k_{12}, and k_{13} in the sketch) are lower than the ones for the second (k_{11}, k_{14}, and k_{18} in the sketch), as metabolites are degraded much faster than proteins. Finally, rate k_{17} represents fructose uptake and was measured experimentally (i.e., q_S). As for the initial values for the state of the promoters in the model (P_1 and P_2), they were estimated from this work, their state with the regulator bound [$P(r)_1$ and $P(r)_2$] was set to be 0 at the beginning of the simulation. Note that neither the free Cra protein nor its repressed complex (Cra^r, bound to F1P) are subjected to degradation rates. This is because they are not subjected to production either; these reactions were deleted for the sake of simplicity (it is just the presence or absence of Cra what matters in the model, and not its production dynamics). The state of all the rest of the individual species was initially set to 0, representing an initial pristine state (see also Table S2 for further details).
