# Supplementary material for: A Metabolic Widget Adjusts the Phosphoenolpyruvate-Dependent Fructose Influx in Pseudomonas putida
Source: mSystems. 2016 Dec 6;1(6):e00154-16. doi: 10.1128/mSystems.00154-16 (PMC5141268; doi:10.1128/mSystems.00154-16)
Supplement: Figure S5 [file sys006162068sf5.pdf]

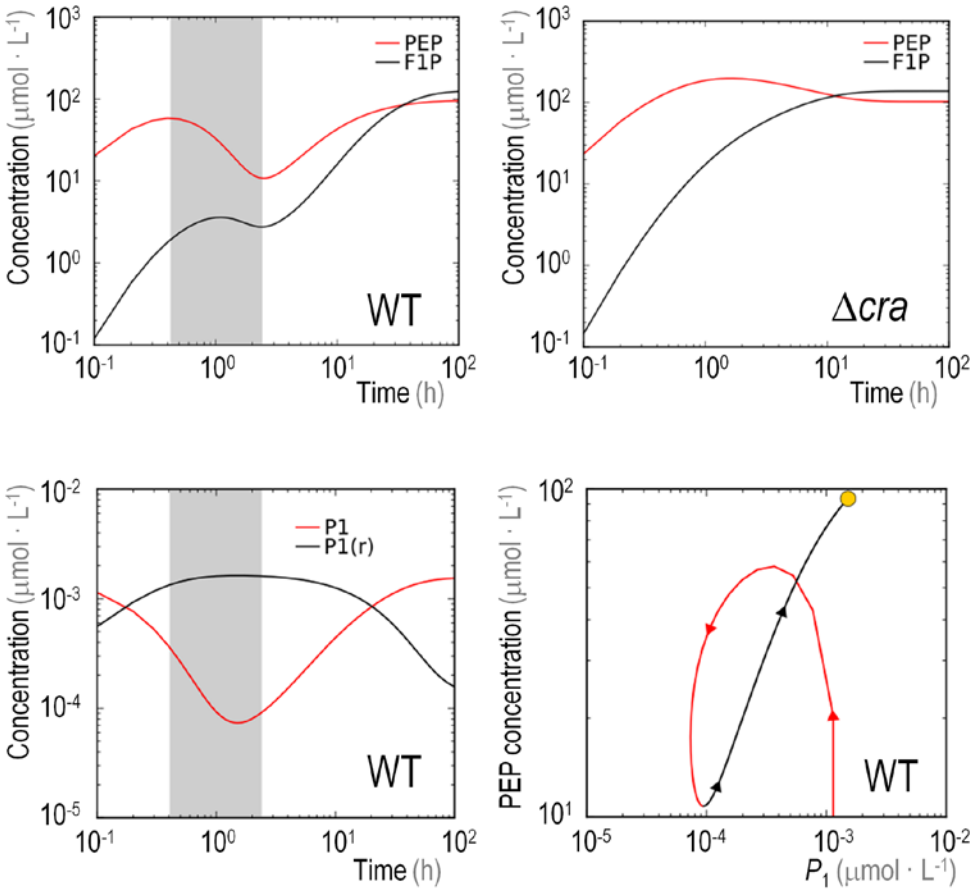

Fig. S5. Dynamic modeling of the intracellular availability of some molecular species in the metabolic widget. *Top row*: phosphoenolpyruvate (PEP) and fructose-1-*P* (F1P) levels over time in the wild-type (WT) cell (*top left*) and in the Δ*cra* mutant (*top right*). The levels at the start/end of the graphs are similar, as observed *in vivo*, but the transitions between different states fluctuate substantially. The instability pointed out by the shadowed region (*top left*) is due to the positive feedback of F1P over PEP *via* the inhibition exerted by Cra$^{P.\ putida}$. Therefore, in the Δ*cra* mutant (*top right*) such an event is not observed. *Bottom left*: The same time-course simulation was repeated but in this case the promoter upstream to *cra* is monitored in its two possible states: P_1, promoter without Cra$^{P.\ putida}$ bound to the corresponding operator, and P_1(r), promoter with Cra$^{P.\ putida}$ bound in the corresponding operator (note that the sum of both states is always constant). *Bottom right*: Interaction loop. Relationship between promoter P_1 (when Cra$^{P.\ putida}$ is not bound to the operator sequence, *x*-axis) and the concentration of PEP (*y*-axis). Initially, P_1 is free of Cra$^{P.\ putida}$ (far right in the *x*-axis) that is why the PEP concentration is rapidly increasing (going up in the *y*-axis). Cra$^{P.\ putida}$ starts repressing P_1 (thus going left on *x*-axis) and, as a consequence, the level of PEP decreases (going down in the *y*-axis). Finally, F1P represses Cra$^{P.\ putida}$, therefore P_1 is free again and the concentration of PEP increases up to the experimentally measured value (yellow dot).
